# Automatic captioning for medical imaging (MIC): a rapid review of literature

**DOI:** 10.1007/s10462-022-10270-w

**Published:** 2022-09-17

**Authors:** Djamila-Romaissa Beddiar, Mourad Oussalah, Tapio Seppänen

**Affiliations:** 1 Center for Machine Vision and Signal Analysis, University of Oulu, 90014 Oulu, Finland; 2 Faculty of Medicine, University of Oulu, 90014 Oulu, Finland

**Keywords:** Automatic image captioning, Caption, Diagnosis generation, Medical images, Rapid review, Report generation, PRISMA

## Abstract

Automatically understanding the content of medical images and delivering accurate descriptions is an emerging field of artificial intelligence that combines skills in both computer vision and natural language processing fields. Medical image captioning is involved in various applications related to diagnosis, treatment, report generation and computer-aided diagnosis to facilitate the decision making and clinical workflows. Unlike generic image captioning, medical image captioning highlights the relationships between image objects and clinical findings, which makes it a very challenging task. Although few review papers have already been published in this field, their coverage is still quite limited and only particular problems are addressed. This motivates the current paper where a rapid review protocol was adopted to review the latest achievements in automatic medical image captioning from the medical domain perspective. We aim through this review to provide the reader with an up-to-date literature in this field by summarizing the key findings and approaches in this field, including the related datasets, applications and limitations as well as highlighting the main competitions, challenges and future directions.

## Introduction

Medical images remain one of the best ways to look inside the body without the need for surgery or other invasive procedures (Allaouzi et al. 2018). They hold pathological information about different organs or tissues (Zeng et al. 2020b) that could be used to diagnose patients and deliver appropriate treatment (Xiong et al. 2019). Recently, with the advances in digital health technology and cloud infrastructure, hospitals constantly produce a large number of medical images generated from different modalities that can be fit for different purposes. However, the task of manually summarizing insights gained from medical images or generating the corresponding reports is very tedious and time-consuming (Zeng et al. 2020b; Ionescu et al. 2017; Harzig et al. 2019; Lyndon et al. 2017). For instance, a radiologist spends 5 to 20 minutes to read, understand and describe the findings of one single CT / ultrasonic image for one patient case (Yin et al. 2019). This was for example observed during the Covid-19 pandemic where radiologists had to read and report more than 100 chest X-rays per day (Monshi et al. 2020) together with a huge increase in hospitals admission rate. This created scenarios where radiologists were unable to provide accurate reports in the required time, which further delayed patients’ stay in hospitals (Han et al. 2021), increased treatment costs, and risk of further spreading the pandemic within the patient community (Han et al. 2018; Mishra et al. 2020; Benzarti et al. 2021). In addition, medical images collected from medical equipment are often limited in scales, heterogeneous, complex and, sometimes, of low quality (Ambati and Reddy Dudyala 2018; Zeng et al. 2020b; Xue et al. 2018). Although we can often easily and directly describe the content of a natural image by observing its content (Zeng et al. 2020b; Sun et al. 2019), the specific characteristics of medical images as mentioned earlier, render the task of generating appropriate medical reports very difficult. This makes it hard to obtain robust models for reasoning.

On the other hand, the reading of even experienced medical professionals (Xu et al. 2019; Lyndon et al. 2017; Gajbhiye et al. 2020) is prone to errors (Ionescu et al. 2020; Wu et al. 2017; Singh et al. 2019), which constitutes a real bottleneck in clinical diagnosis (Ionescu et al. 2017; Yin et al. 2019). As a result, medical imaging analysis is often outsourced (Monshi et al. 2020). This challenge is due to various factors that are rooted back in inherent characteristics of medical images and the requirements of the expected reports. For instance, medical reports should follow specific templates (e.g., boundary conditions and fixed templates) (Li et al. 2018; Wang et al. 2020; Li et al. 2019; Wang et al. 2019) and high level of accuracy when describing the structures, locations, and anatomies, which require extensive expertise (Yang et al. 2021; Allaouzi et al. 2018). Also, medical reports contain coherent paragraphs rather than a set of sentences and should be generated with high precision in practice (Zeng et al. 2020b; Yin et al. 2019; Huang et al. 2019; Singh et al. 2019). They include medical terms that should be generated in a crucial and particular order (Huang et al. 2019; Li et al. 2018; Wang et al. 2020; Li et al. 2019). Moreover, a desirable report should describe normal and abnormal findings and give visual evidences such as location of abnormality and its characteristics (Li et al. 2018; Yin et al. 2019; Ouyang et al. 2020). This should be performed carefully especially that abnormalities are rare and medical reports are mainly dominated by normal findings rather than abnormal results (Li et al. 2018; Yin et al. 2019). In other words, abnormal cases have to be taken into account and described accurately when templates are being produced.

Notably, classical captioning of natural images performs poorly on report generation (Xie et al. 2019; Rodin et al. 2019) due to its domain-specific and language-specific features, as well as the nature of abnormal findings (Xie et al. 2019; Sun et al. 2019; Villegas et al. 2015). To date, the current automated report generation still needs enhancement to be clinically acceptable (Syeda-Mahmood et al. 2020). Therefore, major efforts have been shifted towards initiating new approaches for fast exploitation of medical image content to assist doctors in decision-making (Zeng et al. 2020b; Yuan et al. 2019). This raised the significance of research in automatic captioning of medical images to alleviate the workload of clinicians (Yuan et al. 2019; Yin et al. 2019), deliver faster interpretation of the findings and expedite the clinical workflows (Syeda-Mahmood et al. 2020; Xue et al. 2018).

In general, automatic captioning of images aims to provide a cheap and a meaningful description of the content of the image by retrieving and interpreting its relevant features(Allaouzi et al. 2018). This enables computers to better understand the content of the input images (Allaouzi et al. 2018) and build, accordingly, a bridge to the human world (Zeng et al. 2020b). This helps in improving the qualitative and quantitative assessments of images (Gajbhiye et al. 2020). It can also be used for semantic tagging, image retrieval, image classification, early childhood learning, helping visually impaired persons, human-like robot-robot interactions (Ayesha et al. 2021), visual question answering tasks, and medical diagnosis (Pelka et al. 2019). In essence, automatic Medical Image Captioning (MIC) aims first and foremost at generating accurate, informative, complete, and coherent medical reports from visual observations (Xiong et al. 2019; Yang et al. 2021; Yin et al. 2019). To understand the role of image captioning in the medical field, we attempt through this rapid review to answer three main research questions: *RQ.1:* Is the machine able to accurately and quickly detect and recognize illnesses or abnormalities, and produce informative captions from medical images? (Xiong et al. 2019)? *RQ.2:* Can MIC save labor costs? *RQ.3:* Can MIC compensate for the lack of experienced medical experts?

Automatic medical report generation is regarded as the main application of image captioning models in the medical domain (Yin et al. 2019; Yang et al. 2021). Besides, the generated report is expected to describe the medical image which is similar to the one provided by a professional expert (Shin et al. 2016; Rodin et al. 2019; Monshi et al. 2020). Such automated report could have a great impact in hospitals, in terms of efficiency, accuracy and overall cost saving (Lyndon et al. 2017; Syeda-Mahmood et al. 2020). Similarly, diagnosis-like reports associated with the findings could inform whether the region in the image is normal, abnormal or potentially abnormal (Huang et al. 2019; Harzig et al. 2019), which may accelerate the diagnosis and thus the detection of potentially dangerous diseases. This contributes to early diagnosis and disease screening as well as facilitating the human-machine interactive diagnosis practice (Wang et al. 2019). Furthermore, they can be used in case of emergency when expert doctors are not available, to initiate treatment (Ayesha et al. 2021) and compensate for staff shortages (Xue et al. 2019).

Moreover, automatic reports generation can help to reduce the rate of misdiagnosis and missed diagnosis (Huang et al. 2019; Singh et al. 2019; Pavlopoulos et al. 2021) and quicken the initiation of many specific therapies and treatments (Gajbhiye et al. 2020; Han et al. 2018, 2021). Furthermore, preliminary readings may reduce the burden of clinical report writing and allow the experts to review, edit and improve the final findings (Singh et al. 2019; Park et al. 2020; Hasan et al. 2018) with more relevant details. Many doctors and professionals may often need a second opinion for report writing, which could be helpful through automatic report generation (Singh et al. 2019; Xue et al. 2019). This can greatly contribute to the management of patient care (Tian et al. 2020).

Therefore, automatic medical image captioning helps us to write reports that record the findings on interesting areas of the medical image (Yang et al. 2021) for each patient case. This allows us to describe images with a specific vocabulary and medical lexicon (Alsharid et al. 2019; Kisilev et al. 2015). Overall, image captioning in the medical field can help patients and doctors understand obscured medical images (Zeng et al. 2018; Wu et al. 2017) by directly converting them into text annotations. It is also to note that automatic generation of medical reports can help to unify and improve the quality of generated descriptions which may vary significantly because of the expertise levels of the involved doctors (Yang et al. 2021) when this is conducted manually. In this respect, automatic medical image captioning can play a central role in patient care in general and the application of computer-aided diagnosis (Zeng et al. 2020b; Hasan et al. 2018; Han et al. 2021) or image-assisted diagnosis (Wang 2019) in particular. Computer-aided diagnosis provides accurate diagnosis decisions in a specific lexicon that can be easily understood by experts (Kisilev et al. 2015) and which enables a significant reduction of their workload (Gu et al. 2019; Zeng et al. 2020b).

**Fig. 1:**
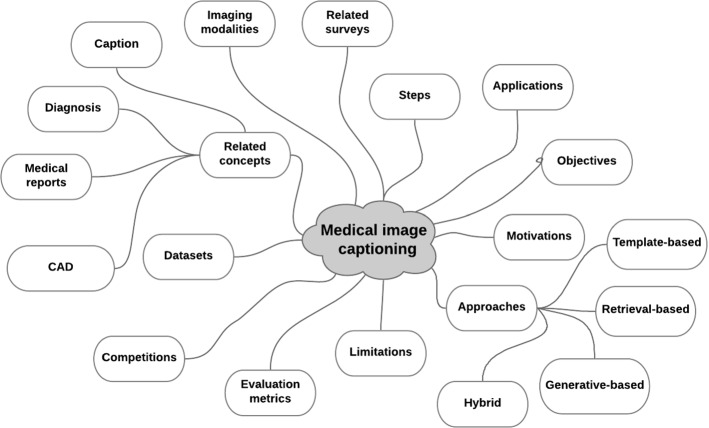
Taxonomy of MIC related aspects discussed in this survey

Several approaches have been proposed in the literature to tackle the task of automatic image captioning. However, only a few techniques were adapted to deal with medical images. This is due, in fact, to both the inherent properties of medical images with their complex anatomy structures and to the fact that the annotations should only focus on clinically important aspects that are relevant to the diagnosis, rather than mapping all objects in the image as in a generic image captioning system (Hossain et al. 2019). An analysis of existing survey papers in this field revealed that they mainly covered some aspects of MIC only, lacking the in-depth analysis of the state-of-the-art techniques. In this respect, the current survey completes and updates the existing surveys. In essence, the current survey differs from existing ones from the following standpoints:

- We discussed the most significant up-to-date advances reported in the literature of MIC, covering concepts related to medical images, their modalities, objectives, application domains, and key motivations that prompted the researchers to investigate this field.
- Unlike (Monshi et al. 2020) who focused on radiology images only, we covered a wide range of other categories, presenting a comprehensive analysis of MIC systems as highlighted in Fig. 1.
- We contrasted commonly employed approaches and extensively discussed their merits and limitations through categorization and exemplification while extending the work reported in (Monshi et al. 2020).
- We considered a categorization scheme that classifies MIC methods according to the architecture used for caption generating into template-based, retrieval-based, generative-based, and hybrid methods.
- We enumerated and reviewed the performance metrics employed in commonly used benchmark datasets of medical image captioning.
- We highlighted existing competitions and challenges related to medical image captioning such as ImageCLEF and provided future directions and useful insights to MIC development community.
- Unlike many other reviews, we detailed and documented the methodology and protocol that guided our literature search.

This paper is organized as follows. First, we provide an overview of MIC-related surveys. Especially, we gathered five surveys and discussed the merits and weaknesses of each one in Section  2. In Sect.  3, we detailed the methodology followed to construct this rapid review using PRISMA’s systematic review protocol. We investigated each step of the PRISMA methodology and discussed its associated output. Next, before going deeper into the MIC analysis, we defined some related concepts and enumerated the attributes constituting typical medical diagnosis reports in Sect.  4. We enumerated as well medical imaging modalities and presented some examples of each modality in Sect.  4.1. Subsequently, we reviewed in Sect.  5.1, the various approaches proposed in the literature to resolve the task of MIC, which we categorize into template-based, retrieval-based, generative-based and hybrid methods. Generative-based methods or deep learning-based methods are further categorized into merge models, encoder-decoder models, and attention-based techniques. Afterward, we enumerated the publicly available benchmark dataset that include medical images and their captions in Sect.  5.2. In Sect.  5.3, we explored the used automatic metrics for performance validation of MIC systems. Next, the limitations of MIC systems are discussed in Sect.  5.4. We enumerated some competitions and challenges dedicated to MIC and included tasks related to MIC in Sect.  6. Finally, we finish with a conclusion where we highlighted some key insights and future directions.

**Table 1:** Analysis of some state-of-the-art comprehensive surveys on MIC

Survey	Year	Image modality	Datasets	Methods	Evaluation measures	Observations
Allaouzi et al. (2018)	2018	No specific modality	IU chest X-ray, PEIR gross, ICLEFCaption, BCIDR	Generative-based models and retrieval-based methods	BLEU, METEOR, CIDer, SPICE, ROUGE	Too short and did not analyze accurately the state-of-the-art
Pavlopoulos et al. (2019)	2019	No specific modality	IU chest X-ray, PEIR gross, ICLEFCaption	Encoder-decoder based methods and retrieval-based methods	BLEU, METEOR, CIDer, SPICE, ROUGE	Did not analyze accurately the literature, missing datasets, metrics, and methods not structured.
Pavlopoulos et al. (2021)	2021	No specific modality	IU chest X-ray, MIMIC-cxr	Early approaches, DL-based generative models, template-based and retrieval-based	Word overlap metrics: BLEU, METEOR, CIDEr, SPICE, ROUGE and clinical correctness measures: keyword accuracy	Not well structured, many aspects related to MIC are missing, few datasets highlighted and study methodology not mentioned.
Monshi et al. (2020)	2020	Radiology	IU chest X-ray, CheXpert, X-ray14, Mimic-cxr, PadChest, PEIR gross, DDSM	DL-based methods	ROUGE, BLEU, METEOR, CIDEr, SPICE and qualitative evaluation	DL-driven review, ignoring other techniques and also emphasis made only on radiology and passing over other modalities.
Ayesha et al. (2021)	2021	No specific modality	ICLEFCaption, BCIDR, IU chest X-ray, X-ray14, PEIR gross, PadChest, CheXpert, Mimic-cxr	DL-based methods	BLEU, METEOR, CIDer, SPICE, ROUGE-L	Focus made upon DL-based method ignoring other techniques.
Ours	2022	No specific modality	ICLEFCaption, BCIDR, IU chest X-ray, PEIR gross, PadChest, CheXpert, Mimic-cxr, ROCO	Generative-based models, retrieval-based methods, template-based methods and hybrid models	ROUGE, BLEU, METEOR, CIDEr, SPICE	Deeper analysis.

BLEU stand for Bilingual Evaluation Understudy score, METEOR for Metric for Evaluation of Translation with Explicit Ordering, ROUGE-L for Recall Oriented Understudy for Gisting Evaluation-Longest common subsequence, CIDer for Consensus-based Image Description Evaluation and SPICE for Semantic Propositional Image Caption Evaluation. For the datasets’ full names, refer to section  5.2

## Related surveys

Despite the recent advances in image analysis, deep learning technology, and natural image captioning (Zohourianshahzadi and Kalita 2021; Srihari 1994), image captioning has not been extensively reviewed in the medical field (Sun et al. 2019). For instance, Hossain *et al.* (Hossain et al. 2019) presented a comprehensive survey on image captioning with an emphasis on deep-learning based methods. However, they did not consider the medical field in their study. Also, (Ghosh et al. 2019) provided an extensive survey on deep-learning based image segmentation techniques, which could constitute a prior step to the image captioning task. The authors discussed medical imaging as well and enumerated some related datasets that could be used for image segmentation. Moreover, several systems and datasets have been released only in the last few years, which call for a constant update on the topic. Besides, surveys on automatic captioning of medical images are still very limited in scope and range. Only five surveys (Allaouzi et al. 2018; Ayesha et al. 2021; Pavlopoulos et al. 2019, 2021; Monshi et al. 2020) have been identified and scrutinized in this section whose summary is provided in Table  1.

In 2018, Allaouzi et al. (2018) provided possibly the first comprehensive review of automatic image captioning in the medical domain. They covered most of the existing methods, benchmark medical image caption datasets and evaluation metrics used in the surveyed literature. Though, the survey was too short and did not analyze the state-of-the-art methods accurately. Later on, Pavlopoulos et al. (2019) proposed another brief overview of biomedical image captioning. The authors discussed the existing datasets, evaluation metrics and some state-of-the-art methods, although, the survey is ill-structured and limited in scope. A more extensive version of this review was presented in 2021 (Pavlopoulos et al. 2021). The latter attempted to discuss the state-of-the-art of diagnostic captioning systems while highlighting relevant publicly available datasets, evaluation measures and future directions in the field. Similarly, Monshi et al. (2020) proposed a comprehensive literature survey on medical image captioning focusing on radiology images and datasets as well as discussing deep learning based approaches for generating radiology reports. They categorized the existing generative approaches into three main levels: words, sentences, and paragraphs. Nevertheless, the survey considered only deep-learning-based approaches for generating diagnostic reports and excluded other techniques and other imaging modalities outside radiology. Finally, we shall mention the comprehensive review of Ayesha et al. (2021). It provided an analysis and a comparison of existing studies on MIC from 2017 to 2020 with a focus on deep-learning based approaches. Publicly available dataset, evaluation measures used for deep-learning based MIC, and future recommendations were further discussed.

The current paper extends the previous reviews by providing a deeper analysis of medical image captioning. Especially, we provide an extensive review of the state-of-the-art approaches which we categorize into four main approaches: template-based, retrieval-based, generative models, and hybrid methods that combine generative deep-learning based techniques and retrieval techniques. Further, we enumerate the benchmark datasets for MIC as well as evaluation metrics. Moreover, we provide the reader with important concepts related to MIC, motivation grounds and key medical applications. Finally, we discuss the limitations and challenges of the current approaches of MIC as well as the key competitions organized in this field.

## Methodology for rapid review-based approach

Boosted by recent advances in deep-learning technology and natural language processing, the field of medical image captioning has seen a renewed interest in recent years where several prototypes have been put forward for automatic report generation from medical imaging. However, many limitations and challenges are still open and many areas can further be exploited. Besides, the diversity of concepts applied in MIC and the growing applications in the field together with the increasing scope of the methodologies employed call for concise and up-to-date surveys in the field. For this purpose, we propose using a rapid review approach to analyze and synthesize existing techniques of automatic medical image captioning in the literature. For that, we followed the PRISMA methodology (Moher et al. 2009) of systematic reviews, which we adopted for a rapid review by reducing the number of databases to be explored and where the grey literature is excluded from the resources. More specifically, we performed the following subsequent steps:

**Table 2:** Search keywords and their substitutes

Keyword	Can be substituted by
Captioning	Caption generation, Report generation, Diagnosis generation, Description, Annotation, Diagnostic captioning
Medical	Biomedical, Ultrasound, MRI, CT, Radiology, PET, XRay
Imaging	Image, Radiograph, Figure
Automatic	Automated

**Fig. 2:**
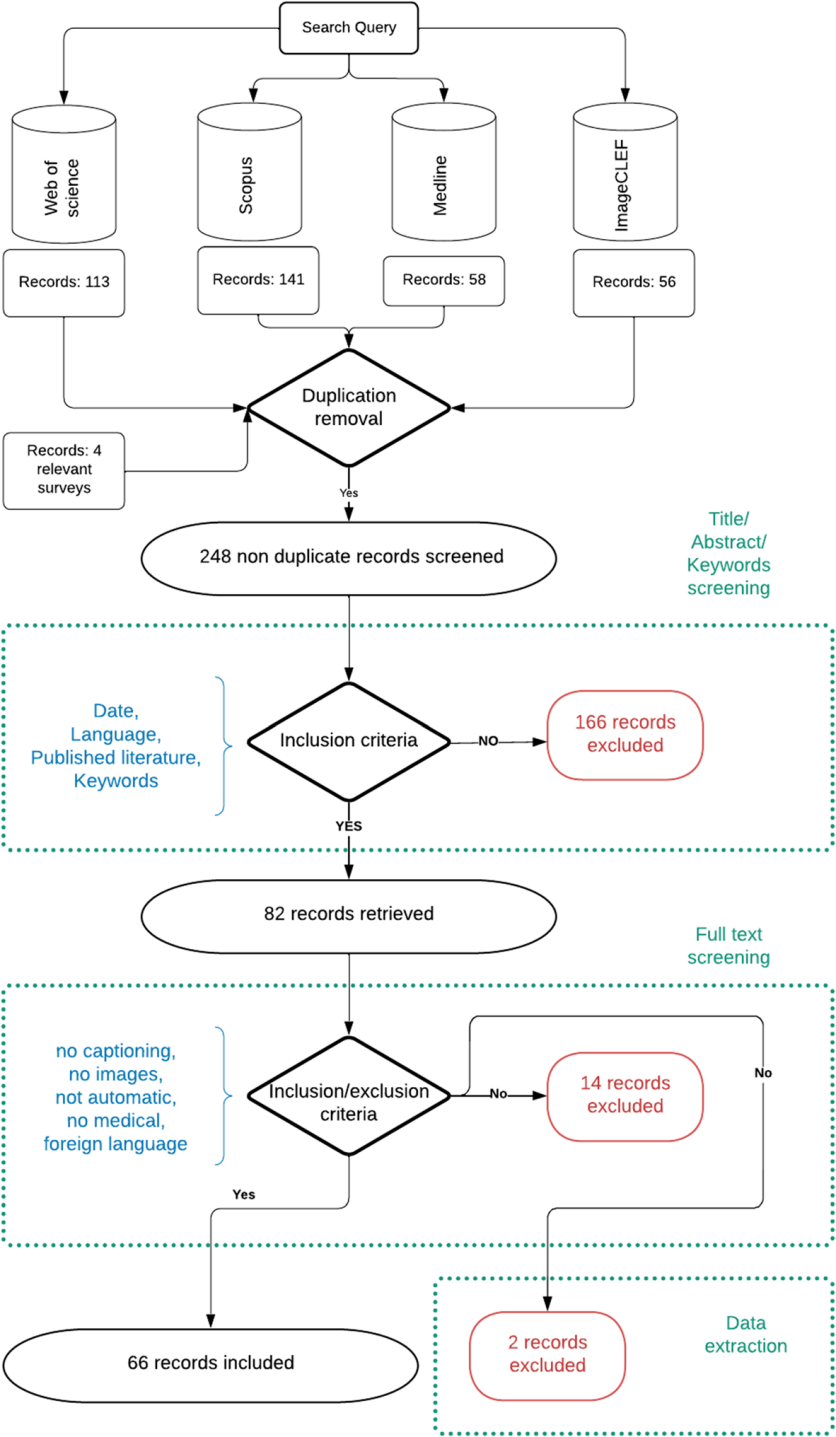
Flow Diagram of our review methodology

1. Question identification: we define the search topic or research question by identifying the list of keywords and their associated substitutes that can help in matching relevant literature in the field of MIC. Table  2 enumerates the list of keywords employed in our search queries. A logical combination (AND or OR) of keywords (or, equivalently, their substitutes) was adopted to construct the query search. These keywords were searched in the title, abstract and keyword list (if available) of the papers retrieved by the search operation.
2. Parameters definition: to obtain relevant results, the following questions are attempted:
  - What resources should be included in our research?
    A rapid review requires few databases compared to a systematic review. So, we investigated three databases: Web of science, Scopus, Medline in addition to the proceedings of the ImageCLEF conferences. We are interested in these databases because of their widespread use in artificial intelligence and medical research communities. Similarly, we investigated the proceedings of the ImageCLEF conferences because they include a particular task related to image captioning and annotation. We used the search API of each database and saved the outputs into files where different elements are taken into account such as title, abstract, keywords, journal name, link in the journal.
  - What are the inclusion/exclusion criteria?
    To include a publication using the above query in the aforementioned databases, we apply specific filters that consist of the following:
    Date: We restrict our research to studies published in the last two decades (2000 to 2021), as the field of automatic MIC is quite recent.
    Language: We restrict our research to publications written in English,
    Source: We only include published literature in the aforementioned databases,
    Keywords: Any publication should include the following keywords or at least one of their substitutes: {automatic, captioning, medical, images}
    Answers that do not match one of the above inclusion criteria are trivially excluded.
  - What is the screening protocol?
    To reduce the risk of bias, we include extensive research outputs which may help us to identify as much relevant literature as possible. We start with review papers and then research papers, and, finally, we search within the reference lists of the included records to identify other interesting resources. For that, we implement the following screening process (given in details in Fig. 2):
    (i) We first merge search results from different sources using appropriate reference management software. For that, we use the screen web application Rayyan QCRI (Ouzzani et al. 2016), and automatically remove duplicate records of the same publication.
    (ii) We examine the titles, keywords and abstracts to remove irrelevant publications as per the previously mentioned inclusion criteria.
    (iii) We retrieve the full text of the potentially relevant publications.
    (iv) We group together multiple publications of the same study, where we create labels for each class of publications.
    (v) We examine the identified full-text publications for compliance of studies with the eligibility criteria.
    (vi) We make the final decisions on study inclusion and proceed to data collection.
    (vii) Finally, we tag or record any ongoing publications which have not yet been reported but included in the reference list of included records, so that they can be added to the ongoing studies table after checking against inclusion criteria as well.
    Besides, we manage alerts for each of the previously searched databases to stay up to date with literature currently being published and keep the review as up-to-date as possible at the time of publication. Moreover, we document the search process and our decisions for all records identified by the research to ensure that this can be reported correctly in the review.
  - How did we appraise the quality of selected studies? What tool/rubric did we use?
    To design a robust high-quality search strategy, it is strongly recommended to peer review each step of the screening process. This helps us to identify relevant studies and to include extensive search outputs. However, in our case only two reviewers conducted the process, and any conflict was resolved through dialogue and communication. We present a flow diagram in Fig. 2, which summarizes the details and the results of our screening protocol.
    To appraise the quality of the selected studies, we use the available tool in (CASP 2021). We present the results of the used checklists in “Appendix. A”.
3. **Biases identification:** As mentioned in the previous step, the quality of our rapid review is appraised using available tools and checklists of the CASP designed for use with systematic reviews (CASP 2021). So, we identified some biases that we enumerate in the following:
  Less transparency and reproducibility with increased errors, due to the fact that only two reviewers performed the screening task and one for the search execution,
  Our study excludes unpublished data since we decided not to include grey literature,
  Our study may exclude significant studies not published in English.
4. Search execution: Once our protocol and methodology are set up, we determine the best method for documenting our search. For this purpose, we select the citation management tool, then we execute the search and store the citation records, for which we use the Rayyan QCRI web application (Ouzzani et al. 2016). In summary, we conducted the screen search based on the inclusion/exclusion criteria that we identified in previous steps. Then, we established a summary matrix, which is briefly presented in “Appendix. B”, to keep track of the screening and review process. Finally, we appraised the quality of the review and presented the results in the next sections of this review.

### Results of the rapid review

At the end of the review, we identified sixty six relevant records that we analyze in this paper.

**Fig. 3:**
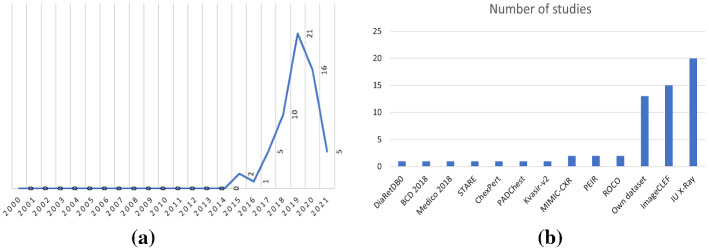
Results of our rapid review **a** Number of records studied per year, **b** Number of included studies that used each of the existing benchmark datasets

We present in Fig. 3a, the yearly evolution of the number of records in the field. It can be observed that from 2000 to 2014, no relevant study was found in the field of automatic medical image captioning. However, from 2015, the number of publications started to increase, especially, in 2019 due to the development of new large scale annotated medical dataset, which stimulated the research in the field and yielded 21 relevant publications.

Moreover, we observe in Fig .3b that the most used dataset in the reviewed studies is the Indiana University Chest X-Ray Collection (IU X-ray dataset). Especially, many publications focusing on chest X-ray images evaluated their findings on this dataset. The dataset presented in the ImageCLEF challenge is another popular resource that has been used in 15 studies. In 13 publications, the researchers proposed their own dataset either by combining medical images from different datasets or by using data recorded from various hospitals, while less popular datasets include ROCO, MIMIC cxr, PEIR, among others.

**Fig. 4:**
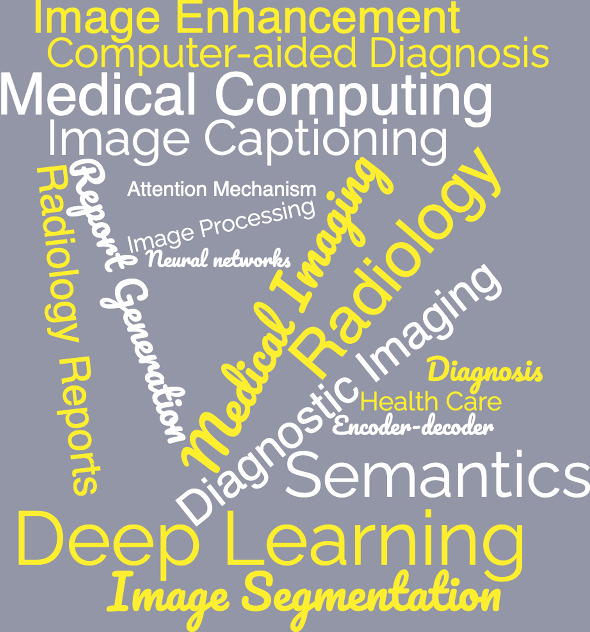
Word cloud for our rapid review results

In addition, we present in Fig. 4 the word-cloud representation of all identified records, highlighting the most commonly used words in all records. We shall observe the dominance of wording associated with medical, images, generation, automatic, reports, diagnosis, annotation, retrieval $\ldots$ etc. The size of the font indicates the frequency of occurrence of each term.

## Definitions and related concepts

To introduce the topic of automatic medical image captioning, it is important to define some related concepts. In this section, we present some useful definitions that help shape the boundaries of the MIC field.

*Caption* is defined in the Cambridge Dictionary as “a short piece of text under a picture in a book, magazine, or newspaper that describes the picture or explains what the people in it are doing or saying”. In Merriam-Webster Dictionary, it is defined as “the explanatory comment or designation accompanying a pictorial illustration”. From the Artificial Intelligence perspective, a caption corresponds to a textual report, regardless of its length, that describes a fine detail of object (s) in the image.

*Image captioning* is defined as the task of generating textual reports, referred to as captions, to describe image content (Alsharid et al. 2019). It involves computer vision for image processing and natural language processing for the caption generation (Alsharid et al. 2019; Allaouzi et al. 2018). Authors of Li et al. (2018), Huang et al. (2019) and Alsharid et al. (2020) defined image captioning as the process of generation of descriptive sentence (s) for images or videos. Benzarti et al. (2021) considered an image as a block of low-level Voxels whose analysis aims to provide a high-level semantic layer by generating a proper annotation. Therefore, image captioning corresponds to an automated objective description of the image that requires a high level of semantic understanding of its content. Describing the spatial regions of an image with multiple captions is known as a dense captioning (Gajbhiye et al. 2020). As a special case, image captioning that uses medical imaging is called medical image captioning (MIC) and is primarily related to diagnosis report generation (Park et al. 2020).

*Diagnostic captioning (DC)* It is defined as the automatic generation of diagnosis from examination of patient medical imaging for diagnosis purposes (Pavlopoulos et al. 2021). The output of DC is communicated to the clinician as supporting evidence that can enhance his diagnosis by highlighting only clinically important information. DC and MIC could be used interchangeably to refer to the same concept.

*Diagnosis report* In the medical field, a report is defined as a detailed account or statement whereas diagnosis refers to the decision reached by the act of identifying a disease from its signs and symptoms. So, a diagnosis report refers to a text-based document elaborated by an expert to describe relevant findings from a medical imaging (Monshi et al. 2020). Notably, this follows critical protocols and uses a particular medical taxonomy that includes visual evidence of findings.

**Fig. 5:**
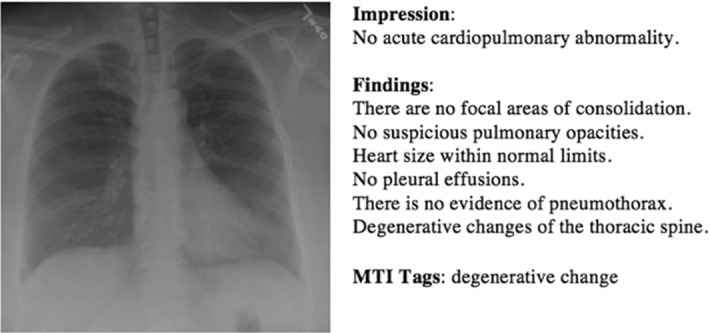
Different sections of the medical reports. Example from the IU X-Ray dataset, retrieved from (Jing et al. 2017)

*Automatic diagnosis report generation* is defined as the task of performing human-like analysis (Rodin et al. 2019) to map visual observations into a set of semantic descriptions (Kisilev et al. 2015). It is a tedious task that allows us to automatically summarize the insights obtained from medical images to construct a relevant diagnosis report (Yin et al. 2019). This is different from image captioning in the sense that (i) medical data is often unavailable; (ii) medical reports are paragraphs rather than sentences; (iii) when used in practice, they should be generated very carefully and with higher precision, and; (iv) medical reports do not focus on objects where nothing clinical is to be reported in the image but tries to cover normal and abnormal findings related to medical attributes (Yuan et al. 2019). (Yang et al. 2020; Li et al. 2019; Pavlopoulos et al. 2021).

In general, medical reports are composed of different sections which we illustrate in Fig. 5 and detail in the following (Xiong et al. 2019; Li et al. 2018):

- *Indication*: consists of a simple statement that provides some historical clinical information about the patient including gender, age as well as the reason for the study;
- *Tags*: consist of pathological keywords which represent the critical information extracted from the findings;
- *Findings*: consist of multiple sentences providing a detailed description of observations regarding different regions in the image, that may help to identify the disease or the abnormality;
- *Impression*: consists of a single-sentence conclusion of the diagnosis which is established from the findings. It may summarize the findings and the patient’s clinical history. At the same time, it is considered to be the most important section of the medical report which is usually investigated by the clinicians (Tian et al. 2020).

Sometimes, another section is also available in the medical report to indicate whether the current imaging study is compared to prior scanning study or not (Singh et al. 2019) and is called the *Comparison Section*.

*Computer-Aided Diagnosis (CAD)* is defined as the diagnosis made by a physician by taking into account the computer output as a second opinion (Doi et al. 1999). This is different from automated computer diagnosis, which is based on computer algorithms only (Doi 2007). Especially, CAD helps to complement the diagnosis made by the physician and assists him in his final assessment (Monshi et al. 2020). This technique has made significant achievements and can be coupled with MIC to generate fully automated reports.

### Medical imaging modalities

Medical images are of different modalities because of the variety of acquisition technologies (Pelka et al. 2018; Gajbhiye et al. 2020; Kisilev et al. 2015; Jayashree Kalpathy-Cramer 2008) such as Computer Tomography (CT), Ultrasound, X-Ray, Fluoroscopy, Positron Emission Tomography (PET), Mammography, Magnetic Resonance Imaging (MRI), Angiography and PET-CT. Each modality has its own characteristics, advantages, and drawbacks. Thus, automatic medical image captioning relies on these different modalities of medical images and specific techniques could be applied to each particular type of imaging. We enumerate below the most common imaging types:

**Fig. 6:**
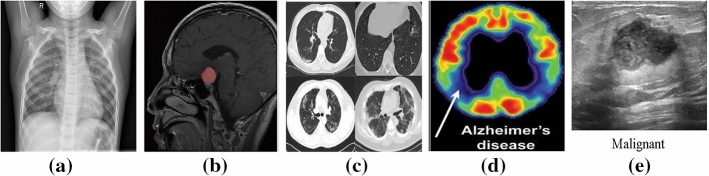
Samples of **a** a normal chest from the Chest X-Ray Images dataset (Kermany et al. 2018), **b** a brain with meningioma MRI Image, retrieved from the Brain MRI Images for Brain Tumor dataset (Cheng 2017), **c** CT scans for COVID-19 patients from the COVID-CT-Dataset (Zhao et al. 2020), **d** PET scans from TADPOLE challenge PET data (Marinescu et al. 2018) for an Alzheimer’s disease and **e** an image from the Ultrasound breast images dataset (Al-Dhabyani et al. 2020)

- *X-ray or Radiography*: is the oldest and the most frequently used imaging technique (Ostensen et al. 2001). It is based on the use of wavelength and frequency of electromagnetic radiation which penetrates the skin and is absorbed by the internal tissues at different rates. A 2D representation of the internal structure is provided by monitoring the variance in absorption (Elangovan and Jeyaseelan 2016). There exist two types of radiography: fluoroscopy and projectional radiography. In addition, radiography is considered low cost, quick, easy to perform but harmful to the human body due to the emitting radiations (Ostensen et al. 2001). An example of an X-ray image of a normal chest from the Chest X-Ray Images dataset (Kermany et al. 2018) is provided in Fig. 6a.
- *Magnetic Resonance Imaging (MRI)*: is employed to visualize detailed internal structures of the body using magnetic radiation (Ostensen et al. 2001). MRI provides a powerful technique that enables multi-planar three-dimensional views of body organs. As we know, the human body is composed of water molecules. When applying a magnetic field, the relaxation of the hydrogen nucleus of the water molecules is exploited and excited (Elangovan and Jeyaseelan 2016). This operation produces a detectable signal that is used to create images, where diverse rates of relaxation of the different tissues allow the identification of potential abnormalities. Unlike CT, MRI does not utilize ionizing harmful radiations, although it is not recommended as a first-stage diagnosis, especially when the patient owns surgical implants (Ostensen et al. 2001). Example of an MRI image is provided in Fig. 6b. Some examples of related works for MIC from MRI images are (Han et al. 2018, 2021).
  The main parts of MRI equipment are a very strong magnet normally in the range of 0.2–2.0 Tesla, a radio transmitter and -receiver, and a computer. The magnet is so large that the patient or the part of the patient to be examined can be placed into it. In that sense, it may look very similar to a CT scanner although the principles for imaging are fundamentally different.
- *Computer Tomography (CT)*: is a new form of X-ray imaging, where a digital reconstruction of images is employed (Elangovan and Jeyaseelan 2016). An X-ray beam is produced by the X-ray tube and goes through the patient body. Then, the detector captures the beam and reconstructs the 2D or 3D images. Each volume of the image is displayed as a pixel encoding the density or attenuation (Ostensen et al. 2001). In addition, contrast media could be employed to distinguish between structures of similar density. Though CT provides detailed images of internal organs, tissues, bones, and blood vessels. It applies high doses of radiation which can be of potential risk to the patient (Ostensen et al. 2001). Samples of CT scans from the COVID-19 dataset (Zhao et al. 2020) are provided in Fig. 6c.
- *Positron Emission Tomography (PET)*: employs a special dye that contains radioactive tracers which are injected into the vein of the examined part of the body. These tracers are then absorbed by certain organs or tissues and tracked by the PET scanner. The latter collects these tracers in areas of higher chemical activity, which promotes the detection of some diseases. Example of a PET image is provided in Fig. 6d from the TADPOLE challenge PET data (Marinescu et al. 2018).
- *Ultrasound*: is a diagnostic imaging technique that uses high-frequency sound waves to examine the internal body structures (Ostensen et al. 2001). Waves whose frequencies are higher than the audio frequency (ultrasound) are sent via conducting gel into tissues with the help of probes. When the waves hit a different structure, they bounce back making it possible to create images (Elangovan and Jeyaseelan 2016). Another type of commonly used ultrasound imaging is Doppler ultrasound which can be used for vascular studies. Ultrasound is cheap and easy to perform, and safe from ionizing radiations (Ostensen et al. 2001). Sample from the Ultrasound breast images dataset (Al-Dhabyani et al. 2020) is provided in Fig. 6e. Some examples of related works for MIC from ultrasound images are Zeng et al. (2020b), Alsharid et al. (2019) and Zeng et al. (2018).

**Fig. 7:**
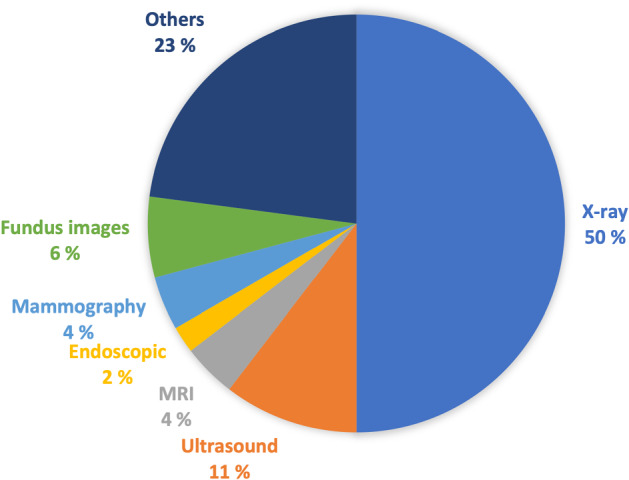
Percentage of included publications in this review according to the imaging modality

As a result of our rapid review, X-ray image modality appears to be the most investigated one in our identified records as shown in Fig. 7. This is likely due to its low cost and easy access. This is also noticed from the fact that the most publicly available and used dataset is the IU X-ray dataset, which has been extensively used for evaluation and comparison purposes by many researchers. On the other hand, the knowledge of imaging modality can be useful to make the captions more focused and accurate.

### Stages of image captioning

In general, image captioning relies on three important steps that apply to all approaches (template-based, retrieval-based or deep-learning-based). We enumerate these steps as follows:

- *Pre-processing*: corresponds to the process of preparing the raw image for the next subsequent tasks. It uses image processing tools to enhance the quality of the image in a way to highlight its various objects relevant for caption generation. A pre-processing step has a profound impact on guiding the output of the caption reports (Ayesha et al. 2021), especially in the case of medical images. Some image augmentation techniques could also be used at this level to increase the size of the dataset.
- *Image feature extraction*: corresponds to the task of identifying and extracting relevant and distinctive features that the image contains. This step is performed using either traditional machine learning tools to extract particular known features or using deep-learning-based models that extract features automatically. It can be followed with a feature reduction method, summarizing, or encoding mechanism to create pertinent features that can be exploited to describe the image. If the used dataset is small, transfer learning is often applied to adjust some large-scale natural image dataset learned features to the target domain.
- *Caption generation*: is the process of translating the extracted features and decoding them into natural language sentences by taking into account the grammatical and semantic aspects that govern the relationships among the identified features. Other approaches consist of either retrieving adequate captions from most similar images whose captions are known or by following a set of defined rules and templates to generate captions from image features.

## Medical image captioning in the literature

In this section, we discuss the existing techniques of MIC in the literature, the commonly used datasets, the performance evaluation metrics as well as the limitations encountered by recent MIC systems.

**Table 3:** Performance results on some state-of-the-art methods for MIC and natural image captioning (rows in italics)

Method	Datasets	B1	B2	B3	B4	M	R	C
Template-based models								
*Kulkarni et al.* (2013)	*PASCAL Sent*	*0.29*				*–*	*0.29*	*–*
Retrieval-based models								
*Farhadi et al. *(2010)	*PASCAL Sent*	*0.76*				*–*	*–*	*–*
Syeda-Mahmood et al. (2020)	Own created	0.56	0.51	0.50	0.49	0.55	0.58	–
Merge models								
Mishra et al. (2020)	Stare	0.87	0.66	0.52	0.44	–	–	–
Alsharid et al. (2020)	Own created	0.27				–	0.42	–
Rahman et al. (2018)	ImageCLEF	0.17				–	–	–
Alsharid et al. (2019)	Own created	0.11				–	0.59	–
Wang et al. (2019)	IU X-ray	0.34	0.22	0.15	0.10	0.14	0.30	0.32
Encoder-decoder models								
* Vinyals et al.* (2015)	*PASCAL VOC*	*0.59*	*–*	*–*	*–*	*–*	*–*	*–*
	*Flickr 8k*	*0.63*	*–*	*–*	*–*	*–*	*–*	*–*
	*Flickr 30k*	*0.66*	*–*	*–*	*–*	*–*	*–*	*–*
	*MSCOCO*	*–*	*–*	*–*	*0.28*	*0.24*	*–*	*0.86*
	*SBU*	*0.28*	*–*	*–*	*–*	*–*	*–*	*–*
*Yao et al.* (2017)	*COCO*	*0.96*	*0.83*	*0.69*	*0.56*	*0.34*	*0.67*	*1.51*
Lydon et al. (2017)	ImageCLEF	0.10				–	–	–
Pelka et al. (2017)	ImageCLEF	0.07				–	–	–
Shin et al. (2016)	IU X-ray	0.78	0.40	0.00	0.00	–	–	–
Zheng et al. (2020a)	Own created	0.63	0.55	0.47	0.42	0.76	–	4.42
Sun et al. (2019)	Own created	0.61	0.41	0.33	0.24	–	–	0.62
Zeng et al. (2020b)	IU X-ray	0.47	0.40	0.30	–	0.45	0.26	3.41
	own created	0.65	0.56	0.45	–	0.45	0.79	4.67
Chelaramani et al. (2020)	Own created	0.32				–	–	–
Zeng et al. (2018)	Own created	0.30	0.22	0.18	–	0.19	0.29	0.99
Haezig et al. (2020)	IU X-ray	0.39	0.27	0.19	0.14	0.18	0.33	0.39
Attention-based Encoder-decoder models								
*Anderson et al.* (2018)	*MSCOCO*	*0.80*	*–*	*–*	*0.36*	*0.28*	*0.57*	*1.20*
*Yao et al.* (2018)	*COCO*	*0.81*	*–*	*–*	*0.38*	*0.29*	*0.59*	*1.29*
*Pan et al.* (2020)	*COCO*	*0.82*	*0.67*	*0.53*	*0.41*	*0.30*	*0.60*	*1.35*
Gajbhiye et al. (2020)	IU X-ray	0.50	0.38	0.32	0.28	0.28	0.44	1.07
Rodin et al. (2019)	Mimic CXR	0.68	0.61	0.54	0.48	–	–	–
Tian et al. (2020)	IU X-ray	0.88	0.87	0.87	0.86	–	0.93	–
Van Sonsbeek et al. (2020)	Mimic CXR	0.36	0.24	0.16	0.093	0.32	0.34	–
Hasan et al. (2018)	ImageCLEF	0.32				–	–	–
Park et al. (2020)	IU X-ray	0.33	0.20	0.14	0.09	–	0.27	0.19
Huang et al. (2019)	IU X-ray	0.48	0.34	0.24	0.17	–	0.35	0.30
Yin et al. (2019)	IU X-ray	0.45	0.29	0.20	0.15	0.18	0.34	0.34
Xiong et al. (2019)	IU X-ray	0.35	0.23	0.14	0.10	–	–	0.32
Yang et al. (2021)	BCD 2018	0.47	0.36	0.27	0.21	0.31	0.46	0.65
Yuan et al. (2019)	ChexPert	0.65	0.50	0.41	0.30	0.42	0.50	–
Yang et al. (2020)	IU X-ray	0.44	0.31	0.22	0.15	–	0.37	0.50
Gu et al. (2019)	own created	0.76	0.72	0.68	0.65	0.49	0.81	–
Xue et al. (2018)	IU X-ray	0.46	0.36	0.27	0.20	0.27	0.37	–
Spinks et al. (2019)	Own created	0.49	0.35	0.25	0.18	0.27	0.40	0.60
Xue et al. (2019)	IU X-ray	0.49	0.34	0.25	0.20	0.23	0.48	0.57
Hybrid models								
Xie et al. (2019)	IU X-ray	0.44	0.34	0.24	0.18	–	0.35	0.37
Wang et al. (2020)	IU X-ray	0.50	0.33	0.24	0.18	–	0.36	0.33
	CX-CHR	0.71	0.64	0.59	0.55	–	0.68	3.25
Li et al. (2018)	IU X-ray	0.44	0.30	0.21	0.15	–	0.32	0.34
	ChexPert	0.67	0.59	0.53	0.49	–	0.61	2.90
Li et al. (2019)	IU X-ray	0.48	0.33	0.23	0.16	–	0.34	0.28
	ChexPert	0.67	0.59	0.53	0.47	–	0.62	2.85

B stands for BLEU, M for METEOR, R for ROUGE-L and C for CIDEr

### Approaches

**Fig. 8:**
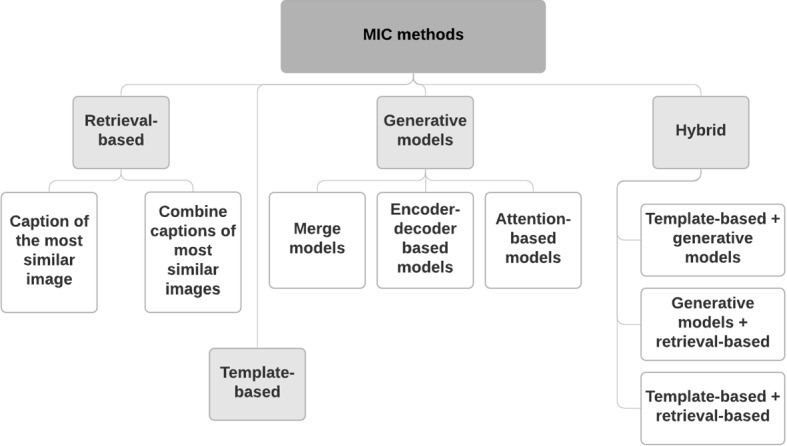
Categorization of MIC methods

With the significant progress of artificial intelligence, different techniques based on machine learning and deep learning have been introduced in the field of image captioning to automatically comprehend the content of the images (Wang 2019). Particularly, many studies focused on describing medical images and interpreting the content of such images to deliver accurate diagnoses and help doctors in the clinical diagnosis workflow (Zeng et al. 2020b). Techniques of automatic captioning of medical images are categorized according to (Allaouzi et al. 2018; Ayesha et al. 2021) into four main classes as illustrated in Fig. 8. We summarize these techniques in Table  3 where we illustrate the performance results on some popular datasets in terms of Bilingual Evaluation Understudy (BLEU) scores, Metric for Evaluation of Translation with Explicit Ordering (METEOR), Recall Oriented Understudy for Gisting Evaluation-Longest common subsequence (ROUGE-L) and Consensus-based Image Description Evaluation (CIDer).

**Fig. 9:**
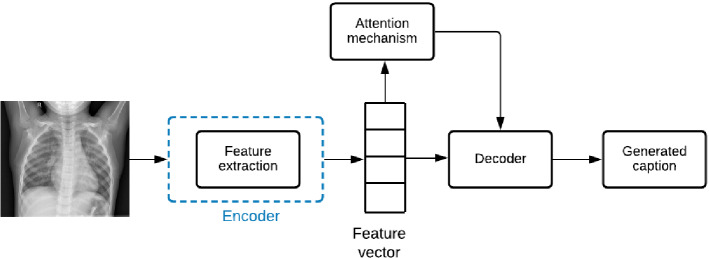
General architecture of Encoder-decoder models with attention

**Fig. 10:**
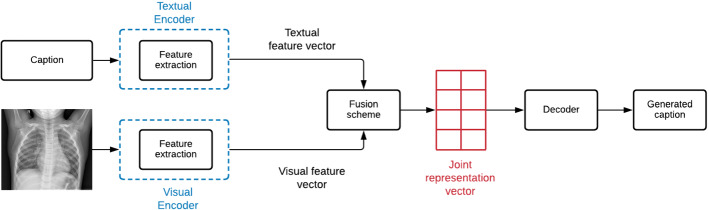
General architecture of merge models (during training phase)

1. *Template-based methods:* These methods rely on the generation of template captions, following some specific rules of grammar. Already available templates are filled out with a specific text describing the findings of medical images. In most cases, when normal results are to be described, it is easier, whereas it is more difficult to fill templates for abnormal findings. Abnormality should be explained and localized in the image and the template should be adapted to include information on such abnormality. This is mainly based on results of object detection and attribute discovery techniques. These methods are simple, and grammatically correct but rely on hard-coded visual concepts, which may constrain the flexibility and the variety of the output (Ayesha et al. 2021). For natural image captioning, Kulkarni et al. (2013) suggested to describe an image by selecting words obtained from statistics mined from visually descriptive text and detection algorithms. They used automatic measures of similarity to compare the constructed sentences from predicted content and natural language general statistics to human-generated reference.
  In the case of medical image captioning, gastrointestinal tract examinations were analyzed by Harzig et al. (2019) to detect diseases and generate descriptive reports from a template library. For that, a deep CNN was proposed to process images extracted from videos and predict their classes while identifying the region that contributed the most to the classification using class activation maps. Similarly, Onita et al. (2020) proposed to map images into a set of words from a dictionary. The proposed technique is based on Kernel Ridge Regression which combines ridge regression and classification. It considers two types of features: RGB pixel values and automatic features extracted with various deep-learning approaches. The main objective of their research was to investigate the influence of the type of text used for describing images (subjective vs objective) as well as the complexity of the deep network used for feature extraction on the model performance. The performance was evaluated on three datasets from different domains including the medical field.
  In addition, Kisilev et al. (2015) proposed a novel method based on lesion boundary detection and image measurements mapped to a set of semantic descriptors aiming at automatically generating breast radiology reports. The CAD system takes into account the relationships between the measurements in a structured learning approach based on an SVM formulation and projects the semantic descriptors into a case reporting. For ImageCLEF 2021, Wang et al. (2021) suggested using pattern-based combination of medical concepts identified in the first task of the challenge to generate new captions.
2. Retrieval-based models: These methods rely on the assumption that similar images have the same captions. So, for each novel image, a set of images visually similar to the query image is retrieved from a large dataset in the first step. Then, two options are made available, either the caption of the most similar image is assigned to the novel image, or candidate captions are exploited and combined to generate a new caption based on some predefined rules and schemes (Ayesha et al. 2021). Farhadi et al. (2010) used score computation to map a sentence to a given image or retrieve images that illustrate a particular sentence. The score is calculated by mapping the image space to the sentence space through some *meaning space*. Similarly, Wang et al. (2019) proposed to combine transfer learning and multi-label classification with an information retrieval-based topic modeling method for the concept detection from medical images. First, concepts are extracted from medical images, then similar images are retrieved using Lucene Image Retrieval (LIRE). Latent Dirichlet al.location (LDA) is used later to analyze the topic distribution of the detected concepts and finally, the topic modelling method is employed to select the most relevant concepts for a given test image. The proposal was submitted to the ImageCLEF caption 2018 task in addition to a newly constructed collection from the ImageCLEF caption 2018 dataset called ImageSem. The authors extended their work and submitted it to the ImageCLEFmed Caption 2019 task. The extension includes a multi-label classification of medical images that have been clustered into different groups based on the body parts in line with their semantic annotated concepts. For ImageCLEF 2021, Charalampakos et al. (2021) proposed a retrieval approach, based on KNN and their previous work (Kougia et al. 2021). Cosine similarity is used to retrieve images from the training images based on the similarity between their embeddings. Captions of most similar images are employed to construct the new caption.
  Syeda-Mahmood et al. (2020) proposed a domain-aware automatic chest X-ray radiology report generation algorithm, that retrieves similar reports from a large database. The retrieval is based on a feature pyramid constructed from coarse and fine-grained descriptions obtained from images, which corresponds to finding labels. Indeed, image features are exploited to extract the finding labels from images.Concept detection and phrasal grouping algorithms are employed to perform automatic labeling, allowing the recognition and the mapping of descriptions to images. Finally, this mapping allows matching images to a pre-assembled database of label patterns and their associated reports which are retrieved accordingly. Also, the authors exploited a visual attention mechanism (Pavlopoulos et al. 2021) to focus on particular vectors of the input text encoding, when generating new words.
3. Generative models or deep neural networks based captioning: These methods rely on end-to-end trainable networks to learn the mapping from images to captions. Different architectures are employed for this category such as encoder-decoder framework which is the most used architecture, fully connected networks, and convolutional networks (Ayesha et al. 2021). These methods can further be categorized into top-down or bottom-up approaches (Alsharid et al. 2019). In the top-down approach, visual features are mapped into textual attributes to describe the content of the image, whereas in the bottom-up approach, each detected object and concept in the image is described with words that are then combined into sentences using language models to generate the whole caption (Alsharid et al. 2019). We quote here the most frequent architectures:
  Encoder-decoder based models: Different configurations of encoder-decoder models have been presented, but the main idea remains the same. In general, a CNN is used as an image encoder to produce a fixed-length vector representation, and an RNN is applied to decode the representation and generate a descriptive caption as shown in Fig. 9 by ignoring the attention mechanism box. For natural image captioning, Vinyals et al. (2015) presented a generative model inspired by deep recurrent models for machine translation called Show-And-Tell. The model is based on a CNN for image encoding and an LSTM for description generation. This contribution was the basis for the implementation of many image captioning systems, including medical image captioning models. Many researchers such as Pelka et al. (2017) and Tsuneda et al. (2021) adapted this model to medical images and the results seem to be promising. In addition, Yao et al. (2017) proposed to integrate attributes to the CNN-LSTM encoder-decoder model for natural image captioning. The model explores both image representations and high-level attributes for image captioning. They explored the inter-attribute correlations in the Multiple Instance Learning framework to learn the attributes. In contrast, authors of Lyndon et al. (2017) and Pelka et al. (2017) proposed encoder-decoder CNN-RNN based models for the medical image caption prediction task of the ImageCLEF 2017. Lydon et al. (2017) employed various imaging modalities and concepts extracted in the concept detection task. Whereas Pelka et al. (2017) extended the Show-And-Tell captioning model, proposed by Vinyals et al. (2015), to generate automatic keywords, which are then used for caption construction. For ImageCLEF 2021, Castro et al. (2021) ranked first in the caption prediction task, by combining deep learning visual encoder, with a traditional classifier of captions that were re-ranked by statistical information obtained from the training dataset.
    In addition, Ambati et al. (2018) proposed a captioning module to resolve the task of visual question answering. The proposed module combines multi-modal embedding from textual and visual features and GRU decoder to generate a sequence of words constituting the answer. The encoder-decoder architecture for caption generation was also adopted for medical retrieval systems to obtain the query terms such as in Benzarti et al. (2021). In the same spirit, Shin et al. (2016) analyzed different regularization techniques to overcome the problem of data bias. They employed a Network-In-Network (NIN) model for feature encoding and compared GRU to LSTM for annotation learning. Wu et al. (2017) explained the abnormal contents in fundus images using an encoder-decoder model aiming at detecting diabetic retinopathy diseases. Region detection and multi-label classification were also explored in the literature to enhance the performance of the captioning task just as in Zeng et al. (2020a). Additionally, Zheng et al. (2020a) used fast RCNN to detect the focus area, capture distinctive features and encode it into a feature vector. Then, a Regional Proposal Network is used to generate region proposals, provide feature maps and classify the disease. Finally, the LSTM decoder receives the feature map and generates annotation text for ultrasound images. Similarly, Sun et al. (2019) proposed to identify lesions in mammography and extract semantic features using an FCN, inline with a multi-label classifier to focus on both the global and local information of medical images.
    Moreover, Zeng et al. (2020b) combined a lesion area detection module and a diagnosis generation module. The detection model employs visual automatic features and pathological information derived from medical images, multi-label classifier and keyword matching. The diagnosis generation module includes a sentinel gate to fuse grammatical information obtained from the object detection model and semantic information extracted from the pathological data to generate accurate reports. Chelaramani et al. (2020) proposed a multi-task approach to identify diseases from fundus images. Multi-label coarse-grained and fine-grained classification is modeled and used to generate the diagnosis based on transfer learning and teacher forcing learning. Similarly, Zeng et al. (2018) proposed a coarse-to-fine ultrasound image captioning ensemble model that allows to identify the organ and the disease and then describe the content of the image using encoder-decoder architecture. The model was coupled with an ultrasound image dataset and data augmentation, using label-preserving transformations, to improve the generalization ability of the encoding model.
    In different works such as Singh et al. (2019), the decoder is implemented in a multi-stage hierarchical manner to translate medical image features into text. Similarly, Haezig et al. (2020) proposed a hierarchical LSTM model to distinguish between normal and abnormal sentences and generate them using a dual LSTM model.
  Encoder-decoder based models with attention: The attention mechanism has proven its promising results in different tasks such as abnormalities classification and localization like in Ouyang et al. (2020). Therefore, it has been included by researchers in the encoder-decoder models to allow them to focus on particular areas of interest as shown in Fig. 9. This may help to obtain more focused captions. To address the problem of natural image captioning, Anderson et al. (2018) proposed a combined bottom-up and top-down attention mechanism that calculates attention at the level of objects and other salient image regions. Faster R-CNN is used to implement the bottom-up attention which represents a set of salient image regions with pooled convolutional feature vectors. Then, by predicting attention distribution over the image regions using task-specific context, the weighted average of the image feature is computed over all regions. In addition, Yao et al. (2018) proposed to explore the semantic and spatial connections between objects of the image to generate reliable captions by proposing a set of salient image regions using Faster R-CNN and building graphs with GCN. After that, the learned relation-aware region representations on each kind of relationship are fed into one individual attention LSTM decoder to generate the sentence. Pan et al. (2020) introduced a novel unified X-Linear attention block for image captioning based on bilinear pooling to capitalize on visual and spatial information. The model captures interactions between the input features and integrates the X-Linear attention blocks into the image encoder and sentence decoder of the image captioning model to leverage higher-order intra and inter-modal interactions.
    In contrast, for medical image captioning, Gajbhiye et al. (2020) proposed to combine context level visual attention and textual attention from different views of X-ray images by learning heterogeneous semantic patterns of the report using a multi-attention encoder-decoder with teacher forcing strategy.
    In addition, a fusion of healthcare data from multiple sources could improve clinical decisions and may reveal entirely new approaches to treating diseases (Tian et al. 2020). Indeed, Rodin et al. (2019) proposed to use multitask CNN-RNN model with attention, which combines the analysis of frontal chest X-ray images with patient’s recorded information. Hence, the pathology, its location, and its severity are described in the medical report. Similarly, Tian et al. (2020) proposed to combine in addition to images and patient’s indication, the doctor’s observation in a multi-task with co-attention approach. They used a hierarchical LSTM to generate topics and decode embedding to generate sentences of the diagnosis and words similar to the sequence input. In the same spirit, Van Sonsbeek et al. (2020) proposed to combine prior patient information and X-ray scans to produce joint features representation. The model employs an attention mechanism on the classification outputs of the joint features representation to generate a diagnosis. Similarly, Hasan et al. (2018) exploited a soft attention-based encoder-decoder model for caption generation where the encoded information fed to the decoder is the output of a modalities classification module. Park et al. (2020) suggested using co-attention and hierarchical LSTM to focus on abnormal findings by combining feature differences between the normal and abnormal cases with visual information and textual information for diagnosis generation. Likewise, Huang et al. (2019) combined X-ray images and background information in a multi-attention-based approach to focus on both spatial information and image’s channel to determine the content and the localization of each entity of the image. The model includes a hierarchical LSTM decoder to generate sentence topics, fuse the background information with word embedding and generate the most appropriate diagnosis word based on the sentence topic and the merged word embedding. Besides, Yin et al. (2019) melded an abnormalities detection module consisting of a deep CNN-based multi-label classification and a hierarchical RNN for long medical annotations generation using an attention mechanism. The model included as well a topic matching mechanism to make generated reports more accurate and diverse and a global label pooling mechanism to deal with multiple abnormalities present in the image. Xiong et al. (2019) exploited a bottom-up attention mechanism based on a DenseNet model pretrained on Chest-Xray 14 dataset to extract visual features of focus areas from medical images. The features are then decoded by a hierarchical transformer based on a self-critical reinforcement learning method to generate reports. An adaptive multi-modal attention network was also proposed in Yang et al. (2021) to describe important local properties in ultrasound images and generate captions based on stored memories in the LSTM decoder. Multi-label classification is introduced to predict essential local properties and generate semantic features that are fused with visual features. Then, they introduced an adaptive attention mechanism with a sentinel gate to control the attention level at current visual features and language model memories when generating the next word.
    Multi-view approach is coupled with an attention-based hierarchical LSTM to generate radiology reports in Yuan et al. (2019) by using multi-label classification and cross-view consistency. Likewise, Yang et al. (2020) utilized, in addition to textual features, frontal and lateral views images to train two identical separate encoders for visual features extraction. Then, the authors used an LSTM with an attention decoder based on a self-critical training with a coverage reward to encourage the model to produce accurate descriptions. Furthermore, Gu et al. (2019) combined multi-label classification using Spatial Regularization Network (SRN) trained on semantic tags related to pulmonary abnormalities with a binary classification of normal and abnormal symptoms and an attention-based mechanism for pulmonary radiology reports generation. In addition, Xue et al. (2018) proposed a multi-modal recurrent model with attention, able to produce justifications for computer-aided reporting systems. Similarly, Spinks et al. (2019) proposed to justify the diagnosis of medical images by using textual and visual evidence from the nearest alternative diagnosis. The model creates an intermediate space between the text and the image and then the text-to-image Adversarially Regularized Auto-encoder (ARAE) model is trained to generate realistic images that mimic the distribution of the training set. At the inference time, the mapping back from the visual input to the intermediate space is performed using a CNN with an attention mechanism and the decoder is used to generate the diagnosis. Besides, due to the lack of large annotated radiology report datasets, Xue et al. (2019) proposed a new method to transfer visual representations learned on small datasets for a report generation task to complement features learned on another large dataset for a classification task. They introduced an encoder-decoder with an attention model in line with feature transfer and feature fusion models for thoracic disease classification. For the ImageCLEF 2017 caption prediction task, authors in Hasan et al. (2017) and Hasan and Farri (2019) proposed to use encoder-decoder frameworks with attention to generate captions for medical images. Both models rely on a deep CNN encoder and attention-based RNN decoder focusing on salient parts of medical images. Furthermore, Xu et al. (2019) proposed, for the concept detection task of the ImageCLEF 2019, two models based on multi-label classification and CNN-LSTM architecture with an attention mechanism to generate appropriate captions, respectively. Tsuneda et al. (2021) used the “Show, attend and tell” model (Xu et al. 2015) by employing ResNet-101 instead of VGG16 and easy data augmentation technique, for medical image captioning for the ImageCLEF 2021. In addition, Nicolson et al. (2021) proposed to divide images into patches and give them to a visual image transformer ViT that acts as an image encoder, and then captions are generated using a self-attention-based PubMedBERT as the decoder. Beddiar et al. (2021) combined a CNN encoder model with an attention-based GRU language generator model for the caption prediction task. Some studies included reinforcement learning to decide when to switch from recycling previous text or generating new text (Pavlopoulos et al. 2021) such as Xiong et al. (2019) and Li et al. (2018).
  Merge models: Merge models were proposed in Mishra et al. (2020) Alsharid et al. (2020), Rahman et al. (2018) and Wang et al. (2021) where CNN networks are used to extract visual features and RNN to learn textual features. Then, visual and textual features are merged to generate relevant captions as illustrated in Fig. 10 which corresponds to the training process. Unlike encoder-decoder based methods, semantic features and visual features are both exploited and fused to obtain a joint representation vector which helps encoding most significant features in the same embedding space. Then, the decoder employs the joint embeddings to generate new captions. In general, different feature fusion techniques could be used to construct the fused vector of features. Moreover, merge models could be seen as a variant of encoder-decoder based models with a feature fusion module. For instance, we can mention Rahman et al. (2018) which was part of the ImageCLEF caption 2018 task and Mishra et al. (2020) that aimed to detect retinal diseases and generate appropriate captions. In addition, Alsharid et al. (2020) put forward a novel curriculum learning approach for second trimester fetal ultrasound image captioning, where text is obtained from audio recordings, and anatomical structure contained in the image is determined using some classification model and a teacher-like learning during the training phase. Also, Alsharid et al. (2019) used full-length second-trimester fetal ultrasound videos and text derived from accompanying expert voice-over audio recordings to train a CNN-RNN merged model. Teacher forcing training consists in using the ground-truth sequences at every step rather than the sequence of newly generated words at previous steps.
    Moreover, Wang et al. (2019) proposed to use image-text joint embedding extracted by a variational auto-encoder model to create medical image semantic association based on medical knowledge bases. Then, the distribution of multi-modal semantic topics of medical images is modeled using the topic model theory. Next, deep fuzzy logic rules were designed according to the diagnosis logic in the medical imaging diagnosis guide for summarizing and interpreting the abnormal appearance in medical images. Finally, they predicted hierarchical image descriptions with an attention mechanism and introduced them to the language generation module for report generation.
4. Hybrid models: Recently, different studies were also conducted on the combination of generative models, retrieval systems and template-based techniques to produce more relevant and accurate reports. For instance, template-based approaches are fused with generative models in Xie et al. (2019) and Han et al. (2018, 2021). Indeed, Han et al. (2021) proposed a human-like neural-symbolic learning framework for spinal medical report generation from MRI images using a unified template to report the findings. The proposed model employs an adversarial graph network for semantic segmentation to detect abnormalities. The generative network integrates a symbolic graph reasoning module and includes prior domain knowledge. Then, symbolic logical reasoning is carried out to perform a causal effect analysis of detected abnormalities through meta-interpretive learning. Likewise, a weakly supervised framework for radiological report generation from lumbar MRI images is proposed in Han et al. (2018). Recurrent Generative Adversarial Network (RGAN) combining a deep Atrous Convolution Auto-encoder (ACAE) and a Spatial LSTM for generative network and adversarial module for discriminative network is proposed for semantic segmentation and radiological classification. The ACAE is used to encode the spinal structures, the LSTM is used for spatial dynamic modeling and the adversarial module is used for correcting the predicted errors and global contiguity. In addition, an unsupervised symbolic program synthesis approach for positional labeling of multiple spinal structures and a symbolic template-based structural captioning module are implemented. Also, Xie et al. (2019) proposed an attention-based framework that generates descriptions for abnormal observations by providing detailed visual evidence through a topic-guided attention mechanism.
  In contrast, authors in Wang et al. (2020), Kougia et al. (2019) and Li et al. (2018) combined generative models with retrieval systems for MIC. For example, Wang et al. (2020) proposed to alternate between template retrieval and sentence generation for rare abnormal descriptions, depending on a contextual relational-topic encoder produced from visual and textual features. The model allows to incorporate semantic consistency of medical terms using a hybrid-knowledge co-reasoning. Furthermore, the AUEB’s NLP group (Kougia et al. 2019) presented different systems for the Image-CLEFmed 2019 Caption task. First, they proposed a retrieval-based model that exploits the visual features to retrieve the k-most similar images with their known concepts based on the highest cosine similarity. The concepts are further combined to predict relevant captions for the input image. A second system was also proposed by implementing the CheXNet with more classification labels. The encoder-decoder model is based on a deep CNN and a feed-forward neural network (FFNN) for multi-label classification. In addition, they suggested combining the above-mentioned systems to create an ensemble model. Scores are computed for each returned concept and combined with the image similarity scores produced by the retrieval model to choose the most similar concepts. Finally, the last system combines a CNN encoder with an FFNN for multi-label classification and a hierarchical LSTM decoder to generate descriptive concepts from medical images. Besides, a novel Hybrid Retrieval-Generation Reinforced Agent (HRGR-Agent) was presented in Li et al. (2018) where a retrieval policy module is implemented to select for each constructed topic whether to generate a new sentence or retrieve a template sentence. The model is updated via reinforcement learning, guided by sentence-level and word-level rewards.
  In the same spirit, researchers combined retrieval-based systems with template-based systems to generate accurate captions for medical images like in Li et al. (2019). The authors suggested to combine prior medical knowledge and retrieval-based methods with modern learning-based methods. The model relies on abnormality graph learning to predict the disease and detect abnormalities, as well as natural language modeling to retrieve text templates based on detected abnormalities. Finally, a paraphraser adapts the templates, enriches them with detail, or corrects false information if any.

**Fig. 11:**
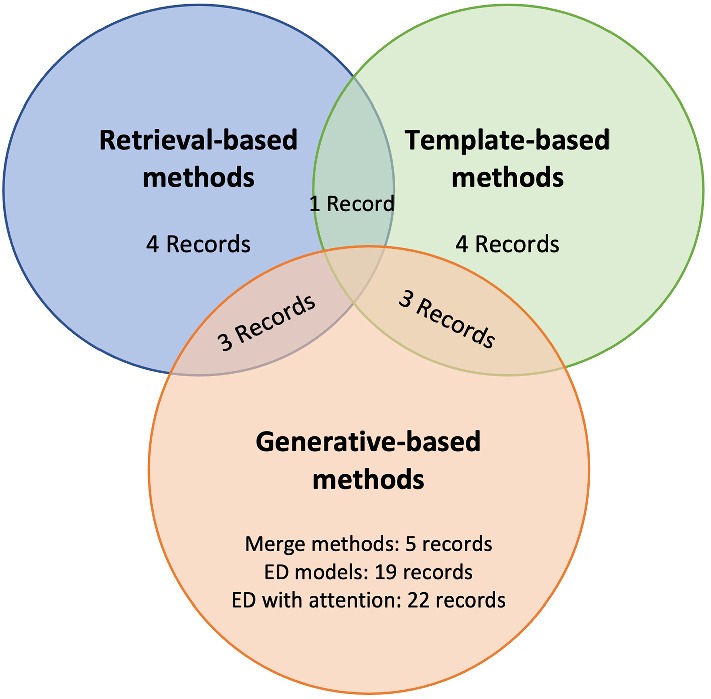
Categorization of studied publications according to the method used for caption generation

Finally, we present in Fig. 11, the number of publications identified in this rapid review and their classification according to the method used for caption generation. We can see from the plot that most methods belong to the generative-based methods. Indeed, 19 records used encoder-decoder architectures whereas 22 included attention mechanisms to focus on interest areas of images, and 5 records combined textual and visual features in a merged architecture. There is also a new trend to combine every two classes of methods like template-based with generative models or generative models with retrieval-based as detailed above. Most notably, attention mechanisms were applied to visual and semantic features to obtain more accurate captions. Generally, most studies used transfer learning to migrate knowledge made in a particular task on a large natural image dataset to a small dataset in the medical domain where relevant medical data is missing. It was observed that template-based and retrieval-based models provided very accurate captions but failed in most cases to report abnormal findings that are novel or rare. It is to note that 5 surveys were analyzed from the total number of 66 records and have not been mentioned in Fig. 11.

### Datasets

To accurately evaluate the performance of the proposed methodologies in different image processing related tasks such as image classification, image retrieval ,$\ldots$ etc, many researchers test their proposals on benchmark datasets. For natural image captioning, there exist many large datasets such as Flickr 8K, TextCaps, COCO. However, datasets with labeled medical images are very limited, which makes the comparison between approaches or the implementation of some deep-learning based approaches unreliable and very restricted. For instance, when dealing with X-ray images, most of the state-of-the-art approaches (Xiong et al. 2019; Yuan et al. 2019; Zeng et al. 2020b; Shin et al. 2016; Harzig et al. 2020; Li et al. 2018; Yin et al. 2019; Huang et al. 2019; Xue et al. 2018; Singh et al. 2019; Gajbhiye et al. 2020; Park et al. 2020; Gu et al. 2019; Xie et al. 2019; Tian et al. 2020; Yang et al. 2020; Li et al. 2019; Xue et al. 2019; Wang et al. 2020, 2019) used the Indiana University Chest X-Ray Collection IU X-Ray (Demner-Fushman et al. 2016), which is a subset of Open-i. Some studies compared the results on the IU X-ray dataset and the CheXpert dataset (Irvin et al. 2019) such as (Yuan et al. 2019; Li et al. 2018). Besides, researchers employed the ImageCLEF dataset when participating in the ImageCLEF competition such as (Pelka et al. 2019; Jayashree Kalpathy-Cramer 2008; Ionescu et al. 2020, 2018, 2017; Villegas et al. 2015; Rahman et al. 2018; Ambati and Reddy Dudyala 2018; Hasan et al. 2018, 2017; Kougia et al. 2019; Xu et al. 2019; Pelka et al. 2017; Ionescu et al. 2019). However, other studies created their own dataset from data available in different hospitals in China, Portugal and USA, like (Gu et al. 2019; Zeng et al. 2020b; Yang et al. 2021; Zeng et al. 2018, 2020a; Alsharid et al. 2020; Han et al. 2018; Kisilev et al. 2015; Sun et al. 2019; Syeda-Mahmood et al. 2020; Chelaramani et al. 2020; Han et al. 2021; Spinks et al. 2019) or from a combination of ImageCLEF datasets with ROCO dataset (Wang 2019). Few works used the PEIR Gross dataset (Yang et al. 2021; Benzarti et al. 2021), the MIMIC-CXR dataset (Johnson et al. 2019; van Sonsbeek et al. 2020; Rodin et al. 2019), the STARE database (Mishra et al. 2020), the Medico 2018 dataset, the Kvasir-v2 dataset (Harzig et al. 2019), the CX-CHR (Li et al. 2019; Wang et al. 2020), the PADChest dataset (Onita et al. 2020) and the database DIARETDB0 (Wu et al. 2017).

We enumerate some commonly used and open-source dataset for medical image captioning in this section and we summarize the most important information on these datasets in Table  4. Nonetheless, we exclude the chest X-ray14 dataset (Wang et al. 2017) since image reports were not available and CX-CHR dataset (Zeiler and Fergus 2014) since it was not open source.

**Table 4:** State-of-the-art dataset, their sizes (# of image-caption pairs), body parts diagnosed, image modality used, source of the images and annotations technique used to create underlying captions. ($\ne$ body parts and $\ne$ modalities mean different body parts and different modalities have been considered by the datasets, respectively)

Dataset	Size	Body part	Images modality	Nature of Images	Annotations
IU Chest X-Ray dataset (Demner-Fushman et al. 2016)	3996	Chest	X-Ray	Real images from the Indiana university hospital	Manual
CheXpert dataset (Irvin et al. 2019)	224,316	Chest	X-Ray	Real images from the Stanford hospital	Automatic
MIMIC-CXR dataset (Johnson et al. 2019)	371,920	Chest	X-Ray	Real images from the Beth Israel Deaconess Medical Center	Automatic
PadChest dataset (Bustos et al. 2020)	160,868	Chest	X-Ray	Real images from the San Juan Spain hospital	Automatic, 27% Manual
BCIDR dataset(Zhang et al. 2017)	5,000	Bladder tissues	–	–	Manual
The PEIR Gross^a^	7,443	$\ne$ body parts	$\ne$ modalities	Pathology Education Informational Resource digital library	Manual
ImageCLEF caption 2017 dataset (Eickhoff et al. 2017)	184,614	$\ne$ body parts	$\ne$ modalities	Open-access biomedical literature database PubMedCentral	–
ImageCLEF caption 2018 dataset (Garcia Seco De Herrera et al. 2018)	232,305	$\ne$ body parts	$\ne$ modalities	Open-access biomedical literature database PubMedCentral	–
ROCO dataset (Pelka et al. 2018)	81,000	$\ne$ body parts	$\ne$ modalities	Open-access biomedical literature database PubMedCentral	–

^a^https://peir.path.uab.edu/library/

**Fig. 12:**
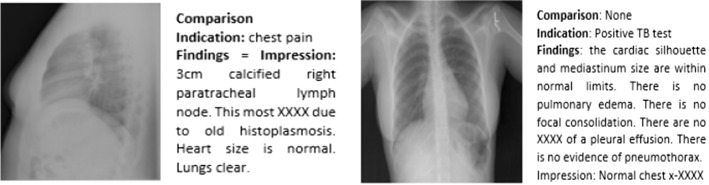
Samples of chest x-ray image-report pairs of two patients and two views (lateral, frontal) from the IU X-Ray dataset (Demner-Fushman et al. 2016)
- *CheXpert dataset *2 Irvin et al. (2019) is a large dataset of automatically annotated chest X-rays images collected from the Stanford Hospital between October 2002 and July 2017 in both inpatient and outpatient centers. It consists of 224,316 multi-view chest radiographs of 65,240 patients labeled for the presence of 14 common chest radiographic observations as positive, negative, or uncertain. The CheXpert labeler was employed to extract annotations from unstructured radiology reports.
- *Medical Information Mart for Intensive Care-Chest X-ray MIMIC-CXR dataset*^3^ Johnson et al. (2019) is one of the latest co-released open source datasets that uses the CheXpert labeler for annotation extraction from radiology reports. It includes 371,920 chest X-rays linked to 227,943 reports that have been gathered from the BIDMC between 2011 and 2016. The reports written in English, were de-identified and images pre-processed to remove any information related to the patient (see Fig. 13).

- *The Indiana University Chest X-Ray Collection IU X-Ray*^1^ Demner-Fushman et al. (2016) is a set of chest x-ray images and their corresponding medical reports provided by the Open Access Biomedical Image Search Engine (OpenI). In total, 3996 radiology reports were collected from the Indiana Network for Patient Care and 8121 associated images from the hospitals’ picture archiving systems (Demner-Fushman et al. 2016). The annotations were performed manually, where the annotators classified and labeled the reports into: normal and subnormal in the first pass. Then, the type of abnormality is used to code the concepts in the second pass for the not normal class. In the end, the dataset contains 7470 images of frontal and lateral X-rays and 3955 reports (see Fig. 12).

**Fig. 13:**
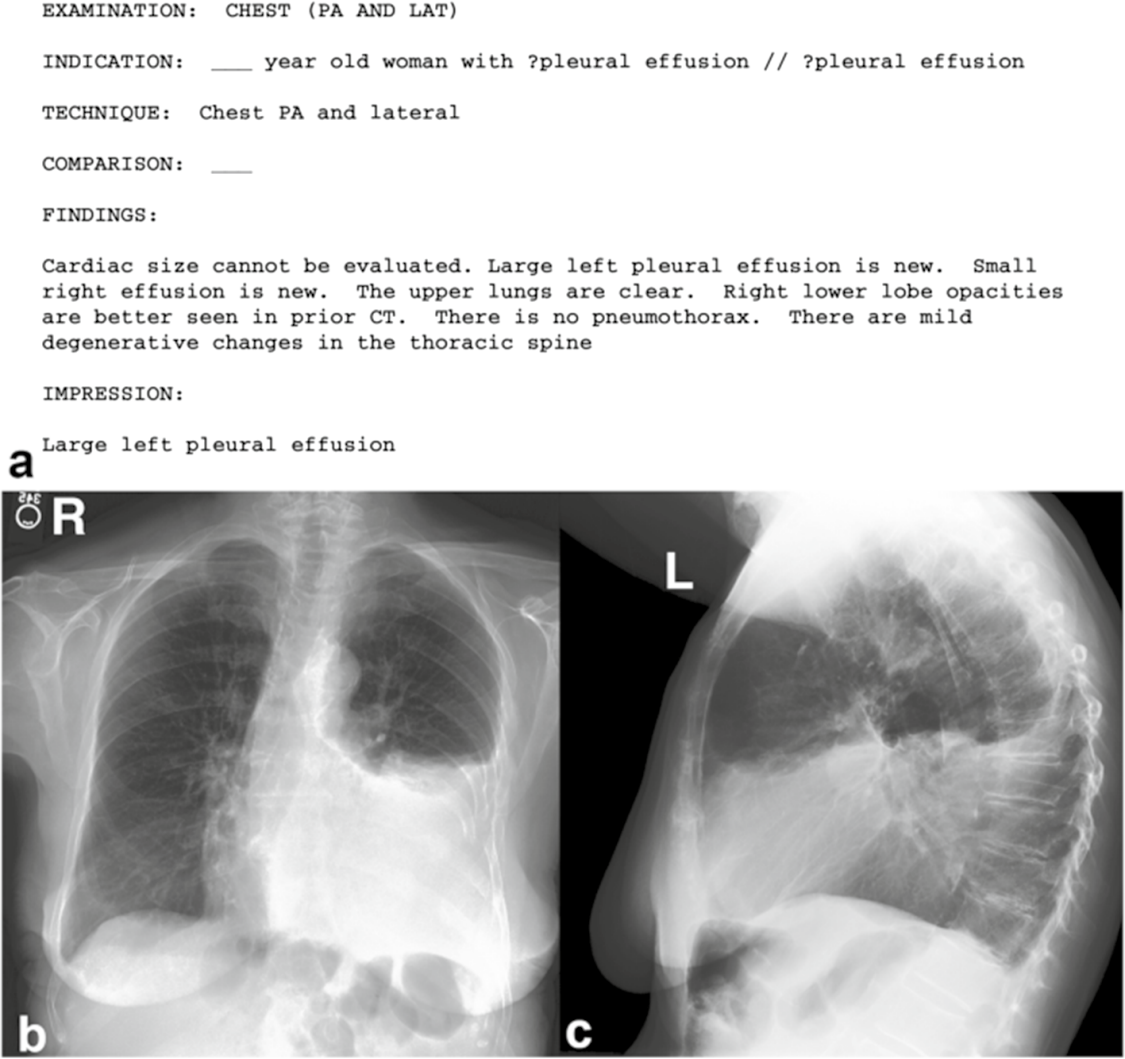
Example study from the MIMIC-CXR dataset. **a** Highlights the radiology report, **b** the frontal view and **c** the lateral view of the chest radiographs
- *Pathology Detection in Chest radiographs PadChest dataset *4 Bustos et al. (2020) is a publicly available dataset that was collected from 69,882 patients at the Hospital San Juan, Spain between 2009 and 2017. It includes 160,868 chest x-rays from 6 different views associated with 109,931 Spanish reports. 27% of the annotations were performed manually by expert physicians while the rest is performed using a supervised method based on a recurrent neural network with attention mechanisms.
- *BCIDR *5 was created by Zhang et al. (2017) is based on image-reports pairs of pathological bladder cancer. Whole-slide images from bladder tissue were taken from 32 patients at risk of a papillary urothelial neoplasm. A subset of 1,000 images was randomly selected and described by a pathologist while addressing 5 types of cell appearance (state of nuclear pleomorphism, cell crowding, cell polarity, mitosis, and prominence of nucleoli) and a conclusion of four classes (normal tissue, low-grade carcinoma, high-grade carcinoma, and insufficient information) is finally derived. Four more descriptions of each image were also provided by doctors leading to 5,000 image-text pairs in total.
- *The Pathology Education Informational Resource PEIR Gross* available at the PEIR Digital Library^6^ is a subset of 7443 images with their captions from 10,000 curated pathology teaching images, stored since 1999. The total database contains 23 sub-categories but only 22 contain a gross sub-collection. It was used first time by Jing et al. (2017) for medical image captioning and each sentence caption contains a single descriptive sentence. This database is provided by Pathology Education Informational Resource digital library for use in medical education and it contains two other sets of images: PEIR Radiology and PEIR Slice (see Fig. 14).

**Fig. 14:**
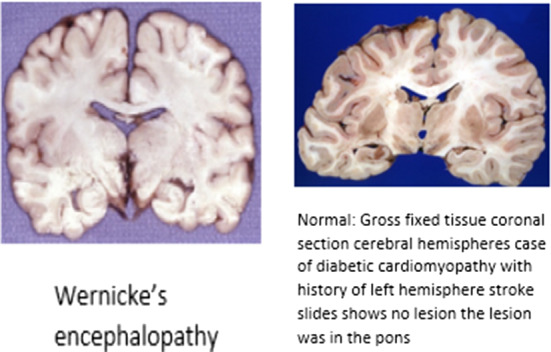
Samples of image-caption pairs from the PEIR Gross subset from the nervous class
- *ImageCLEF image-caption datsets extracted from PubMed Central (PMC)*
  1. ImageCLEF caption 2017^7^ Eickhoff et al. (2017) was created for the Image Concept detection and Caption prediction tasks of the ImageCLEF competition 2017. It contains 184,614 biomedical images, the corresponding UMLS concepts and their captions retrieved from scholarly biomedical articles on PubMed Central (PMC). The dataset is divided into three subsets: (training with 164,614 images, testing and validation with 10,000 images each).
  2. ImageCLEF caption 2018^8^ Garcia Seco De Herrera et al. (2018) is an extension of the ImageCLEF caption 2017 dataset, which is created by classifying 5.8 million images of PMC, using a fully automated deep multi-modal fusion of CNNs. The collection comprises 232,305 image-caption pairs (training with 222,305 images, testing with 10,000 images).
- *ROCO dataset*^9^ was created by Pelka et al. (2018) for multi-modal image captioning. It was constructed by retrieving all image-caption pairs from the open-access biomedical literature database PubMedCentral, eliminating irrelevant images using a binary radiology and non-radiology classification. The dataset contains 81k radiology images with several medical imaging modalities and was used for ImageCLEF 2015 Medical Classification, ImageCLEF 2013/2016 Medical Task.

### Evaluation metrics

To evaluate the performance of the generated captions for natural image captioning, several metrics have been implemented. The same metrics can also be applied to medical image captioning. In fact, they allow us to compute the similarity between the ground truth and the newly generated captions to appraise the quality of the model in constructing new captions. Besides, most studies compared their results to previous state-of-the-art baselines using these metrics (Yuan et al. 2019; Xie et al. 2019; Gu et al. 2019; Kisilev et al. 2015; Park et al. 2020; Singh et al. 2019; Syeda-Mahmood et al. 2020).

Roughly speaking, BLEU, METEOR, CIDEr, and ROUGE-L are the most commonly used automatic metrics for MIC that compute the word overlap. Most of the state-of-the-art techniques that we analyze through this rapid review employed one, two or all of them such as in (Xiong et al. 2019; Alsharid et al. 2019; Zeng et al. 2018; Shin et al. 2016; Yuan et al. 2019; Zeng et al. 2020b; Sun et al. 2019; Huang et al. 2019; Harzig et al. 2020). Besides, F_1 score was also used in different works like (Ionescu et al. 2020, 2019, 2017; Hasan et al. 2018, 2017; Kougia et al. 2019). Other metrics have also been adopted for different approaches, namely: the WBSS (Word-based Semantic Similarity) used in (Ambati and Reddy Dudyala 2018), ROC-AUC score (Rodin et al. 2019; Li et al. 2019), the Euclidean distance (Onita et al. 2020), keywords accuracy which refers to the ratio of the number of keywords correctly generated by the model to the number of all keywords in the ground truth findings (Xie et al. 2019; Xue et al. 2018; Wu et al. 2017) and the anatomical description metric ARS which estimates the matching between generated words and terminology of the anatomical class (Alsharid et al. 2020, 2019; Li et al. 2018).

**Table 5:** Main symbols and notations used for the different evaluation metrics

Symbol	Description
c	Candidate sentence
$r_j$	Reference sentence
$S_r$	Set of reference sentences
N (by default = 4)	The number of n-grams (uni-gram, bi-gram, 3-gram and 4-gram)
$m_r$	Number of words of a given reference sentence
$m_c$	Number of words of the candidate sentence
$u_r$	Number of sentences in the set of reference sentences
R	Recall
P	Precision

**Table 6:** Symbols and notations of equations related to the BLEU metric

Symbol	Description
BP	Brevity penalty
$W_n$ (by default = 1/N = 1/4)	The weight of each modified precision
$p_n$	The modified precision
$CC_N$	Clipped n-gram counts of the candidate sentence in the corpus
$C_N$	The number of candidate n-grams

**Table 7:** Symbols and notations of equations related to the ROUGE-L metric

Symbol	Description
LCS	The longest common subsequence
$P_{lcs}$	LCS-based precision
$R_{lcs}$	LCS-based recall
$\beta$	$P_{lcs}$/$R_{lcs}$
$l_{LCS}$	Length of the longest common subsequence of X and Y
$LCS\cup (r_j,c)$	LCS score of the union longest common subsequence between a reference sentence and the candidate sentence

In addition to the quantitative evaluation (automatic measures), other researchers employed qualitative evaluation. They conducted human evaluation by selecting samples from the testing set and requesting expert evaluation (Yang et al. 2020; Li et al. 2019). Generated captions are judged in terms of language fluency, grammar faultlessness, content coverage, and the correctness of medical concepts. The researchers were able to exploit the Python NLTK, the Python scikit-learn library, and the COCO-caption evaluation API for default implementation of the aforementioned metrics. In addition, the available tools could be used for conducting surveys and human evaluation tasks such as the Amazon Mechanical Turk (MTurk). We quote in this section, the frequently used metrics. For each metric, we introduce the main symbols and notations employed for its calculus in a table to clarify the expressions (Table 5).

**Fig. 15:**
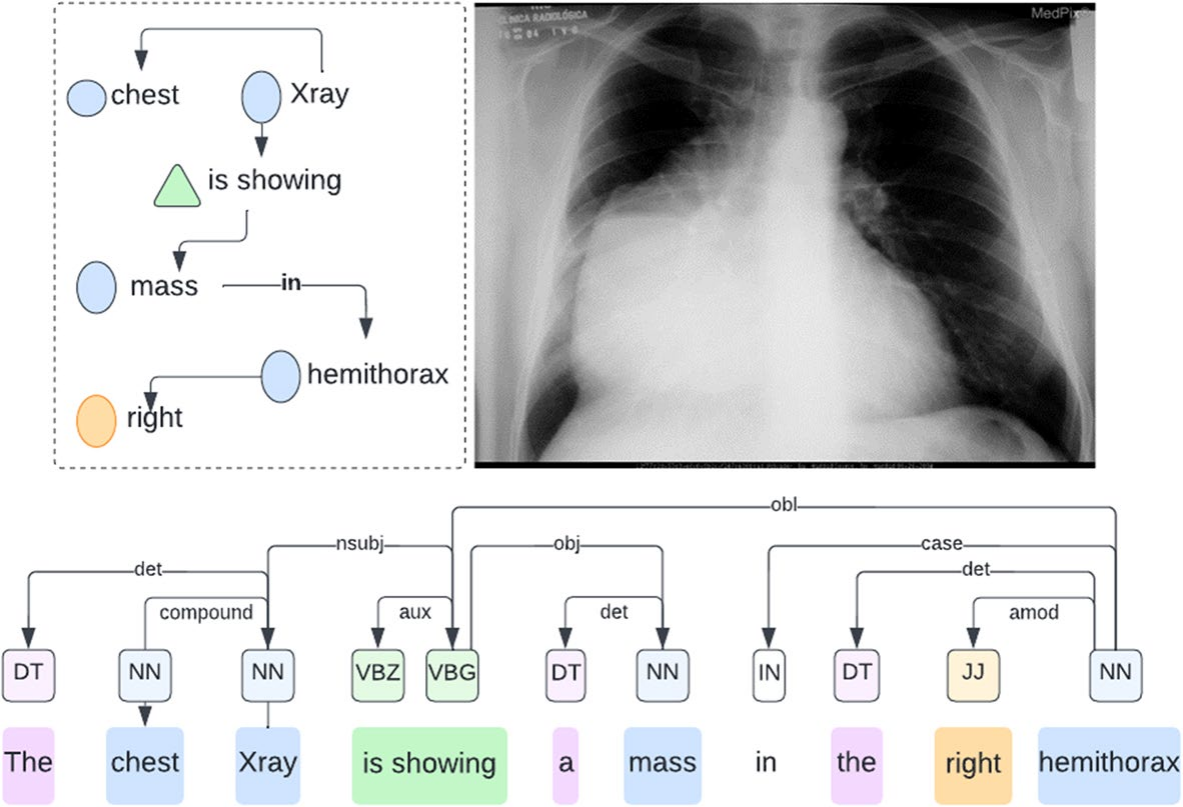
Example of a medical image, its caption, for which part of speech tags (POS tags) and relations between them are shown, and its scene graph. We present the objects (nouns) in blue, the attributes (adjectives in orange) and relationships between objects in green. POS tags are: DT for determiner, NN for nouns, VBZ and VBG for verbs, IN for preposition and JJ for adjective. For dependencies between words, we have det for determiner, compound for compound words, nsubj for nominal topic, aux for auxiliary, obj for object, obl for indirect nominal, case for case marking and amond for adjectival modifier

- *Bilingual Evaluation Understudy BLEU *(Papineni et al. 2002) is a quick, language-independent, automatic machine translation evaluation. BLEU can also be used for text summarization, image captioning, and speech recognition. It allows us to measure the closeness of candidate translation (machine translation) to the reference translation (human translation) taking into account different parameters (word choice, word order, and translation length). BLEU attempts to calculate the position-independent matches between the n-grams of the candidate and those of the reference translation. The machine translation is considered better when the number of matches is greater (BLEU close to 1).
  Mathematically, BLEU computes the precision by clipping which refers to the precision for a word based on the maximum of its occurrences in any reference sentence (Table 6). BLEU can be expressed with:
  1 $$\begin{aligned} BLEU = BP.\mathrm {e}^{\sum _{n=1}^N w_n.\log (p_n)} \end{aligned}$$
  The brevity Penalty (BP) allows us to pick the candidate translation which is most likely close in length, word choice, and word order to the reference translation. It is an exponential decay and is calculated as follows:
  2 $$\begin{aligned} BP = {\left\{ \begin{array}{ll}1 &{} m_c>m_r\\ \mathrm {e}^{(1-m_r/m_c)} &{} m_c \leqslant m_r\end{array}\right. } \end{aligned}$$
  Modified precision is computed for each n-gram as the sum of clipped n-gram counts of the candidate sentences in the corpus $CC_N$ divided by the number of candidate n-grams $C_N$ as shows (3) (Papineni et al. 2002). It allows us to compute the adequacy and the fluency of the candidate translation to the reference translation.
  3 $$\begin{aligned} p_n= \frac{\displaystyle \sum \limits _{C\in \lbrace Candidates\rbrace }\displaystyle \sum \limits _{n-gram\in {C}}CC_N}{\displaystyle \sum \limits _{C'\in \lbrace Candidates\rbrace }\displaystyle \sum \limits _{n-gram'\in {C'}}C_N} \end{aligned}$$
- *Recall Oriented Understudy for Gisting Evaluation-Longest common subsequence ROUGE-L *(Lin 2004) is an automatic evaluation metric that can be used for machine translation and text summarization. It is based on the computation of the longest common subsequence LCS, which refers to the longest matching sequence of words between the original summary and Predicted summary. Using LCS helps us to compute the in-sequence matches that reflect the sentence level word order rather than consecutive matches of words. Another advantage is that LCS automatically includes in-sequence common n-grams, so no need to calculate the predefined sequence of n-grams (Table 7). Mathematically, ROUGE-L could be given by:
  4 $$\begin{aligned} F_{lcs}= \frac{(1+\beta ^2). R_{lcs}.P_{lcs}}{R_{lcs}+\beta ^2 .P_{lcs}} \end{aligned}$$
  The LCS-based precision $P_{lcs}$ and the LCS-based recall $R_{lcs}$ could be computed for a sentence level (upper part of the following equations: (5) and (6)) or summary level (bottom part of equations: (5) and (6)).
  5 $$\begin{aligned}&P_{lcs}= {\left\{ \begin{array}{ll}\frac{l_{LCS}(X,Y)}{m_c} \\ \frac{\displaystyle \sum \limits _{j=1}^{u_r} LCS\cup (r_j,c)}{m_c} \end{array}\right. } \end{aligned}$$
  6 $$\begin{aligned}&R_{lcs}= {\left\{ \begin{array}{ll}\frac{l_{LCS}(X,Y)}{m_r} \\ \frac{\displaystyle \sum \limits _{j=1}^{u_r} LCS\cup (r_j,c)}{m_r} \end{array}\right. } \end{aligned}$$
- *Metric for Evaluation of Translation with Explicit Ordering METEOR *(Banerjee and Lavie 2005) was developed to evaluate the correlation between generated translation and human translation at a sentence level. METEOR computes the F-measure based on an explicit uni-gram matching (word to word matching) between the candidate and the reference translations, and the maximum score is returned (Table 8).
  Mathematically, METEOR is given by:
  7 $$\begin{aligned} METEOR = F_{mean}.(1-pn) \end{aligned}$$
  To compute the penalty *pn*, chunks are composed of unigrams which are adjacent in the hypothesis and in the reference. The longer the adjacent mappings between the candidate and the reference, the fewer chunks there are. The penalty is obtained by:
  8 $$\begin{aligned} pn = 0.5* \left(\frac{Ch}{Um} \right)^3 \end{aligned}$$
  F_mean is calculated as a harmonic mean of precision and recall, where more weight is placed on recall as follows:
  9 $$\begin{aligned} F_{mean} = \frac{10.P.R}{R+9.P} \end{aligned}$$
  The recall value R is obtained using (10) and the precision P is obtained using (11).
  10 $$\begin{aligned}&R = \frac{M(c)}{U(r)} \end{aligned}$$
  11 $$\begin{aligned}&P = \frac{M(c)}{U(c)} \end{aligned}$$
- *Consensus-based Image Description Evaluation CIDEr *(Vedantam et al. 2015) is an automatic consensus-based evaluation metric designed mainly for image description evaluation. It measures the similarity of machine-generated sentence to a set of human descriptions by taking into account grammar, saliency and accuracy (Table 9).
  So, CIDEr$_n$ score for n-grams of length n is computed using the average cosine similarity between candidate sentence and reference sentences as follows:
  12 $$\begin{aligned} CIDEr_n(c,r_j) = \frac{1}{u_r}\displaystyle \sum \limits _{j=1}^{u_r} \frac{g^n(c).g^n(r_j)}{\parallel g^n(c)\parallel .\parallel g^n(r_j)\parallel } \end{aligned}$$
  The TF-IDF weighting $g_k(r_j)$ for each n-gram $w_k$ of a reference sentence is given by the following (similarly for $g_k(c)$, for the candidate sentence, by replacing $r_j$ with *c*):
  13 $$\begin{aligned} g_k(r_j)=\frac{h_k(r_j)}{\displaystyle \sum \limits _{w_l \in \Omega } h_l(r_j)}\log (\frac{|I|}{\displaystyle \sum \limits _{I_p \in I} min(1,\displaystyle \sum \limits _{j} h_k(r_j))}) \end{aligned}$$
  Finally, CIDEr is computed by combining the scores from n-grams of varying lengths as follows:
  14 $$\begin{aligned} CIDEr(c,r_j) = \displaystyle \sum \limits _{n=1}^N w_n.CIDEr_n(c,r_j) \end{aligned}$$
- *Semantic Propositional Image Caption Evaluation SPICE introduced by *(Anderson et al. 2016) is used for caption generation evaluation. SPICE has been used for natural image captioning and could be adopted for MIC as well and this is why we mention it here even though none of the analyzed papers for MIC has employed it for performance evaluation. It is based on the use of a semantic representation by exploiting the scene graph which is obtained using a dependency parser and a rule-based system. The former allows us to establish syntactic dependencies between words in the caption while the latter helps us to map the dependency trees into graphs. In general, the scene graph encodes objects, attributes and relationships between words of the image’s caption. So, candidate and reference captions are first mapped into scene graphs (G(c) and G($S_r$) respectively) where G(c) is given by $G(c) =\langle O(c),E(c),K(c)\rangle$. In addition, G($S_r$) is formed by the union of scene graphs ($\bigcup G(r_j)$) of all reference sentences $r_j$.
  Then, F-score is computed based on the conjunction of logical tuples representing semantic propositions in the scene graph (Table 10). Thus, SPICE is given mathematically by:
  15 $$\begin{aligned} SPICE(c,S_r) = F_1(c,S_r) = \frac{2.P(c,S_r).R(c,S_r)}{P(c,S_r)+R(c,S_r)} \end{aligned}$$
  Where precision and recall are computed with:
  16 $$\begin{aligned}&P = \frac{\mid T(G(c)) \bigotimes T(G(S_r))\mid }{\mid T(G(c)) \mid } \end{aligned}$$
  17 $$\begin{aligned}&R = \frac{ \mid T(G(c)) \bigotimes T(G(S_r)) \mid }{ \mid T(G(S_r)) \mid } \end{aligned}$$
  Each tuple contains either one, two or three elements. E.g. a 3-tuple is ($o_1,e_1,k_1$) while a 2-tuple is ($o_2,e_2$) from a scene graph *G*(*x*). By definition, the function *T* is given by:
  18 $$\begin{aligned} T(G(x))= O(x) \cup E(x) \cup K(x) \end{aligned}$$
  Once all the tuples from both scene graphs (*G*(*c*) and *G*(*S*)) are obtained, the binary matching operator $\bigotimes$ is employed to extract matching tuples from them (E.g. one can use the wordnet synonym matching approach of METEOR (Denkowski and Lavie 2014) for that). In addition, tuples in the same wordnet sysnet or having equal lemmatized word forms are considered to be matched (Anderson et al. 2016).
  We present in Fig. 15, a medical image and its corresponding scene graph. The parts of speech are extracted from the image caption to identify the objects, attributes and relations as shown in the lower part of the figure. Objects in this example are: {chest, Xray, mass, hemithorax}; attributes are: {right} and relations could be identified with the verbs and the corresponding preposition: {is showing, in}. Tuples are then constructed from these sets (objects, attributes and relations). Some examples of tuples are: “Xray”,“mass”,“hemithorax”,“show”,“Chest, Xray”,“Xray, mass”,“show, mass”,“mass, hemithorax”,“right, hemithorax”,“Xray, show, mass”,“show, mass, hemithorax,”,“Xray, mass, hemithorax”,“Xray, show, mass, hemithorax”, “chest, Xray, show, mass, hemithorax”. Finally, the scene graph is drawn from the tuples as illustrated by the left part of the figure. The same process is done for the candidate caption and the reference captions. Union of tuples from the latter is performed and a scene graph is constructed for the set of reference captions. At the end, graph of the candidate caption is matched to the graph of the set of reference captions to identify matching tuples and calculate SPICE.

**Table 8:** Symbols and notations of equations related to the METEOR metric

Symbol	Description
*pn*	Penalty
*Ch*	Number of chunks
*Um*	Number of unigrams that matched between the candidate and the reference
M(c)	Number of unigrams in the candidate sentence that are mapped
U(r)	Total number of unigrams in the reference sentence
U(c)	Total number of unigrams in the candidate sentence

**Table 9:** Symbols and notations of equations related to the CIDEr metric

Symbol	Description
$g^n(c)$	Vector formed by all n-grams of length n of the candidate sentence
$\parallel g^n(c)\parallel$	Magnitude of the vector $g^n(c)$
$g^n(r_j)$	Vector formed by all n-grams of length n of the set of reference sentences
$\parallel g^n(r_j)\parallel$	Magnitude of the vector $g^n(r_j)$
$g_k(r_j)$	TF-IDF weighting for each n-gram $w_k$ of the set of reference sentences
$g_k(c)$	TF-IDF weighting for each n-gram $w_k$ of the candidate sentence
$h_k(r_j)$	Number of occurrences of an n-gram $w_k$ in a reference sentence
$h_k(c)$	Number of occurrences of an n-gram $w_k$ in the candidate sentence
$\Omega$	Vocabulary of all n-grams
I	Set of all images of the dataset

**Table 10:** Symbols and notations of equations related to the SPICE metric

Symbol	Description
G(c)	Scene graph of the candidate sentence
$G(r_j)$	Scene graph of each reference sentence
$G(S_r)$	Scene graph of all reference sentences
O(c)	Set of objects in the candidate sentence
E(c)	Set of attributes in the candidate sentence
K(c)	Set of relations in the candidate sentence
*T*	The function that allows us to return logical tuples

### Limitations

Though many approaches for automatic captioning of medical imaging have been introduced in the literature, they are still limited due to different factors. We enumerate some of these limitations in this section as follows:

1. Related to deep learning based models:
  CAD systems need large amounts of descriptive annotations and should be used carefully to generate proper reports (Han et al. 2018). However, only few large-scale medical image benchmark datasets with captions are available (Alsharid et al. 2019; Allaouzi et al. 2018; Zeng et al. 2018; Yuan et al. 2019; Han et al. 2021; Monshi et al. 2020).
  Most of the existing datasets are small and restricted to one modality of imaging (Rodin et al. 2019), which influences the prediction when using another modality and inhibits the model from generalizing.
  Current models focus on extracting global features and could not localize the abnormality in the image which is mostly available in a particular region of the image and could only be characterized by local features (Yin et al. 2019; Xie et al. 2019; Ambati and Reddy Dudyala 2018). In addition, existing datasets are class imbalanced and abnormal cases are much less than normal cases whereas some abnormalities appear too rarely in the dataset. This influences the reliability of the training model and the detection of rare diseases because the model cannot create a sentence that has never appeared in the training (Park et al. 2020; Wu et al. 2017).
  Most generated reports are constructed using RNN architectures which may greatly suffer from gradient vanishing when the sentences are too long (Xiong et al. 2019).
  Many existing techniques fail in generating words of the sentence in a correct order (Gajbhiye et al. 2020; Zeng et al. 2020b) and current metrics are still not able to capture the change of meaning in the sentence when a punctuation or a negation is present (Singh et al. 2019; Zeng et al. 2020b; Xue et al. 2019; Pavlopoulos et al. 2021).
  Difficulty of evaluation and comparison of newly proposed methods with existing state-of-the-art methods as well as the adoption of deep learning models that require large labeled data are still burdensome (Zeng et al. 2018; Sun et al. 2019; Han et al. 2021; Monshi et al. 2020; Ayesha et al. 2021).
  Even though using transfer learning to overcome the problem of small data is useful, the degree of domain transfer from natural images to medical images is very large and may result in different levels of errors (Lyndon et al. 2017).
2. Related to template-based methods:
  The generated medical image reports should depict some important local properties (e.g., boundary condition, tumor morphology $\ldots$.etc.) and should follow specific templates resulting in fixed phrases and terminology (Yang et al. 2021). In general, they include four parts: indication, tags, findings and impression, which makes the report generation task very challenging, time-consuming (Yuan et al. 2019), and non-trivial (Xiong et al. 2019).
  Specialists often write reports with various styles leading to incoherent labeled data (Han et al. 2021) and may provide different reports to the same image (Xue et al. 2019).
3. General limitations (could be applied to different categories of methods):
  The existing medical data (especially extracted from the scholarly biomedical journal articles) is heterogeneous, noisy and low of quality (Ionescu et al. 2017; Kougia et al. 2019; Park et al. 2020; van Sonsbeek et al. 2020; Xue et al. 2018) and in most cases not real (Pavlopoulos et al. 2021).
  Obtaining medical data could be subject to privacy concerns as they may include personal data of patients (van Sonsbeek et al. 2020) and may lead to incomplete reports (Monshi et al. 2020).
  Constructing datasets using crowd-sourcing is not an option in the medical field since the terminology used to generate medical reports is very precise, heterogeneous, and different than natural language (Allaouzi et al. 2018; Xiong et al. 2019; Yin et al. 2019; Zeng et al. 2020b) and it may lead to propagation of errors from the construction task to the learning process (Hasan et al. 2017). Annotation of medical images is also prone to human errors (Yuan et al. 2019).
  The change in the view of acquisition could influence marginally on the detection of the abnormality because some regions may not be observed and the different views of images should only provide extra information rather than being used for diagnosis (Yuan et al. 2019; Jayashree Kalpathy-Cramer 2008).
  Errors and incoherent sentences (Harzig et al. 2020) or long sentences that do not accurately describe the content of the image (Pelka et al. 2017), are not yet clinically acceptable since they can be very misleading (Yuan et al. 2019; Syeda-Mahmood et al. 2020).
  Existing models still need to include human evaluation (Park et al. 2020; Pavlopoulos et al. 2021) to assess their performance and this remains challenging due to the difficulty and costs of obtaining evaluators with sufficient expertise.
  For the multi-label classification models, it is important to pay attention to the similar appearance of objects to be labeled and to objects that should be detected from sequences of images rather than still images such as the abnormal heart beating motion (Alsharid et al. 2019). Also, recognizing low-frequency concepts or out-of-vocabulary concepts (Wang 2019) is very difficult.
  Dealing with particular image modalities such as ultrasound images is challenging because content analysis and understanding - used to depict disease information - is a relatively rough task and requires deep expertise and experience (Zeng et al. 2018).

## Challenges and competitions

There exist a few challenges and competitions related to automatic captioning of medical imaging. We mention for instance ImageCLEF, which is an evaluation campaign organized each year as part of the CLEF initiative labs. It includes different tasks specific to multimedia retrieval, annotation, and indexing suggesting novel challenges and benchmarking resources (Kougia et al. 2019). Each year, many participants around the world are welcomed to publish innovative proposals based on provided data.

In 2015, Liver CT annotation task was introduced aiming at proposing a computer-aided automatic annotation of liver CT volumes for application as an automated structures report generation (Villegas et al. 2015). Even though 51 online registrations were performed, only one group submitted the results and participated in this task. The task aimed at predicting missing radiological annotations in liver CT image reports (Villegas et al. 2015). So, the participants had to fill structured reports generated using ONLIRA ontology which was enriched to include patient information and was called LICO. In 2016, the medical task ImageCLEFmed (García Seco de Herrera et al. 2016) focused on labelling and separation of compound figures from biomedical literature. A caption prediction subtask was proposed aiming at automatic captioning of medical imaging for diagnosis purposes.

Then, in 2017, in addition to the image caption prediction, another subtask was proposed as concept detection as part of the biomedical image captioning task (Ionescu et al. 2017). The concept detection aimed at retrieving relevant clinical concepts from medical images (Hasan et al. 2017), whereas the caption prediction consisted in generating coherent captions for medical images using concepts retrieved in the first subtask (Pelka et al. 2017). Similar to 2017, there were two main tasks in 2018: concept detection and caption prediction. The former aimed at extracting the main biomedical concepts (e.g., anatomy, finding, diagnosis) from images based only on their visual contents (Ionescu et al. 2018) using Unified Medical Language System (UMLS). However, the last consisted in outputting a human-readable and concise textual description of figures retrieved from biomedical journals based on concepts detected in the first task and visual information captured from the image (Rahman et al. 2018). Moreover, data was modified to respond to some difficulties encountered in 2017. Only two groups participated in both tasks and multi-label classification and retrieval-based approaches dominated the solutions (Ionescu et al. 2018).

Similar to 2018, concept detection and caption prediction tasks were proposed as the third edition in 2019 (Hasan and Farri 2019). However, the first task focused on UMLSR concepts in radiology images only towards automatic medical image captioning and medical reports generation (Kougia et al. 2019). Moreover, the task involved different medical imaging modalities without targeting a particular disease or anatomic structure (Ionescu et al. 2019).

For the 4$^{th}$ edition of the caption prediction task, UMLSR concepts are detected from radiology images including several imaging modality information. However, in 2020, the additional label information is included (modality technique) for pre-filtering and fine-tuning approaches (Ionescu et al. 2020). Transfer learning, RNN and CNN were dominating techniques in this edition. Finally, for ImageCLEF 2021, the focus lies in using real clinical radiology images annotated by medical doctors. The 2021 edition consists as well of two sub-tasks that are similar to previous editions. It involves as well data from the ROCO dataset.

In addition, some different events and conferences focus on image analysis and understanding such as CVPR, ICPR and ACL. Researchers provide each year valuable contributions to these conferences that are sometimes linked to automatic image captioning.

## Conclusion and Future Directions

Automatically understanding and describing the content of medical images is becoming a very promising trend in the medical field. This task is highly correlated to medical image captioning that can be very useful in diagnosis, treatment, surgery and generally, expedition of clinical workflows. This paper aims to give a rapid review of the recent progress made to date in this field of research. It initially presents concepts related to MIC, including imaging modalities, medical report sections and stages of image captioning. Then it discusses the different applications of image captioning with focus on the medical field and the major objectives intended by MIC systems. Afterwards, it highlights the motivations that promoted the research in this field from different perspectives. Subsequently, the current paper analyzes the existing approaches in the literature, which are categorized into four main classes: template-based, retrieval-based, generative models and hybrid techniques and discusses their limitations. Commonly used dataset as well as performance evaluation metrics are also provided and explained. Finally, frequently organized competitions in the field are introduced.

During this study, it is observed that, despite the progress made in image captioning in the literature, its application in the medical field is still very challenging. This is due to the nature of medical images and reports which are different from natural images and generic captions. Indeed, captioning generic images consists in describing the objects and the relationships between them using one or more sentences. However, captioning a medical image consists in understanding the clinical finding and providing an accurate report composed of different paragraphs to highlight only what is clinically important rather than what exists in the image in terms of objects. Also, existing approaches still suffer from certain limitations that we discussed previously. A new trend of hybrid approaches, combining generative and retrieval-based models, seems very promising. Additionally, the need to develop real medical image datasets is increasing with the use of deep-learning methods since they require huge amounts of annotated data. In addition, construction of dataset that include images of different body parts such as brain and breast $\ldots$ etc, is required for further enhancement of image captioning tasks. Furthermore, appropriate domain-specific evaluation metrics have to be put forward to deal with medical report generation since current metrics are not accurate. Again, involving the physician in the report generation could be very beneficial by allowing him to see, correct and approve the automatically generated report and providing him with evidence about each highlighted finding. Increasing the human interaction could also be useful at the stage of accuracy evaluation by incorporating manual evaluation by qualified physicians for better reports. As a conclusion, we can say that the developed techniques in the field of medical image captioning are still facing several problems, and still require demanding challenges to be addressed. We summarize some of these limitations in Table  11 and we provide some potential solutions for each of them. We categorize them into three main classes: Data issues, Model issues and Evaluation issues. For data issues, it is important to mention the availability of balanced MIC datasets which can be used to train deep learning based models. To solve this issue, construction of large labeled and balanced datasets with different data modalities is essential. In addition, privacy issues and anonymization of patients’ data have to be taken into account. The particular nature of medical data and the specific terminology required for medical reports force the implementation of domain-specific models able to deal with discrete features of the medical images and compatible with tailored pre-processing tools. These models should allow the involvement of physicians for more credible and accurate captions. Adopting one method or the other depends marginally on the quality of the generated captions, this is why it is worth considering appropriate domain-specific evaluation metrics. Moreover, explainable AI solutions combined with manual evaluation by qualified physicians seem to be very helpful to understand the results of complex deep learning models and facilitate the process of evaluation.

**Table 11:** Summary of main limitations of MIC systems and potential associated solutions

	Limitations	Potential solutions and ways forward
Data issues	Few, and small MIC dataset with particular image modality and difficulty for generalization of the developed systems	Construction of large scale dataset of real medical images of various body parts and from different modalities.
	Class imbalanced dataset and rarity of abnormal data	Data augmentation for abnormal class expansion, which takes into account the nature of medical images and preserves their contents.
	Lack of resources for use of complex deep learning models and transfer learning	Construction of large scale labeled datasets and intelligent features selection when transferring knowledge from any domain to the medical domain.
	Privacy issues for acquiring medical data	Advanced anonymization and data preprocessing could be implemented to hide patient identity and personal information. In addition, federated learning could be investigated to hide raw data and hence ensure privacy of medical data.
Model issues	Complex nature of medical images that require deep expertise and extensive experience	Domain-specific generative or retrieval based models to deal with discrete features of the medical images. Also, Promoting tailored preprocessing tools for medical images to simplify interpretability.
	Different styles and templates and specific terminology for medical reports generation in addition to human errors	Implementation of unified templates with specific terminology to improve the quality of the generated reports. Also, involving the physician in the process of report generation to correct and approve the automatically generated sentences.
	Incoherent sentences and incorrect order of words generated automatically, which are not clinically acceptable	Involving the physician in the report generation by allowing him to see, correct and approve the automatically generated report and providing him with evidences about each highlighted finding. In addition, the use of specific and limited vocabulary in the generation process could help to provide coherent and efficient reports.
	Difficulty to distinguish purely local features from purely global features	Datasets should include annotated bounding boxes for abnormal regions to allow the system to get fine-grained features (Singh et al. 2019; Ayesha et al. 2021) and precisely mark out the abnormality. In addition, effective attention mechanisms are required to focus on local important regions.
Evaluation issues	Lack of efficiency of evaluation metrics	Development of appropriate domain-specific evaluation metrics to deal with medical report generation.
	Lack of consensus among clinicians on reporting	Promote standards and template reporting in different image modalities and diagnoses. Also, promote the use of explainable AI solutions, which highlight approximations and visualization of complex deep learning models to ease understanding of the results in a way to promote consensus among clinicians.
	Ambiguity and incorrect detection of objects from medical images	Increasing the human interaction by incorporating manual evaluation by qualified physicians for better reports.
	Increasing costs of human evaluation and annotations	Development and implementation of specific crowd-sourcing tools in addition to human-like evaluation mechanisms for medical domain use. This would allow generation of automatic annotations for medical images and facilitate the process of evaluation.

## Figures and Tables

**Fig. 16:**
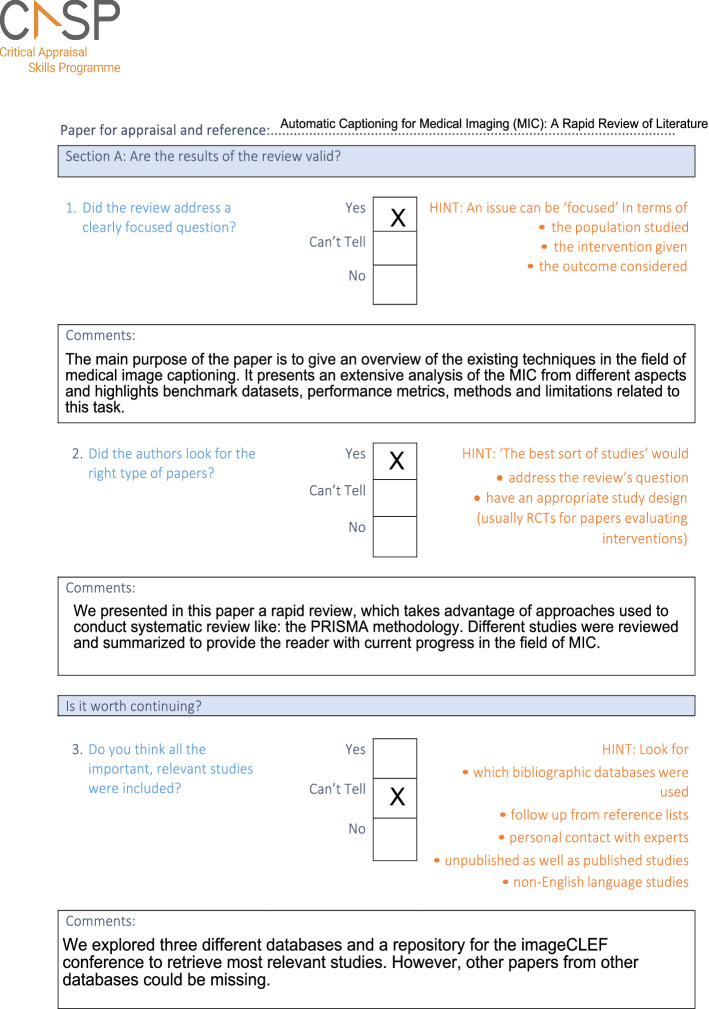
CASP Systematic review Checklist (page1)

**Fig. 17:**
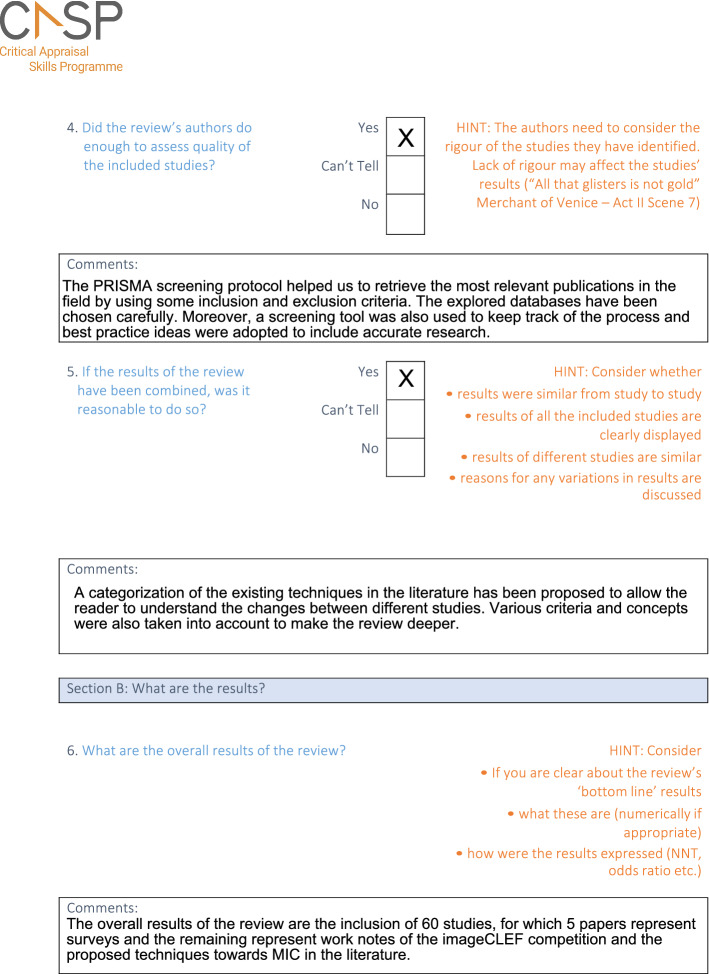
CASP Systematic review Checklist (page2)

**Fig. 18:**
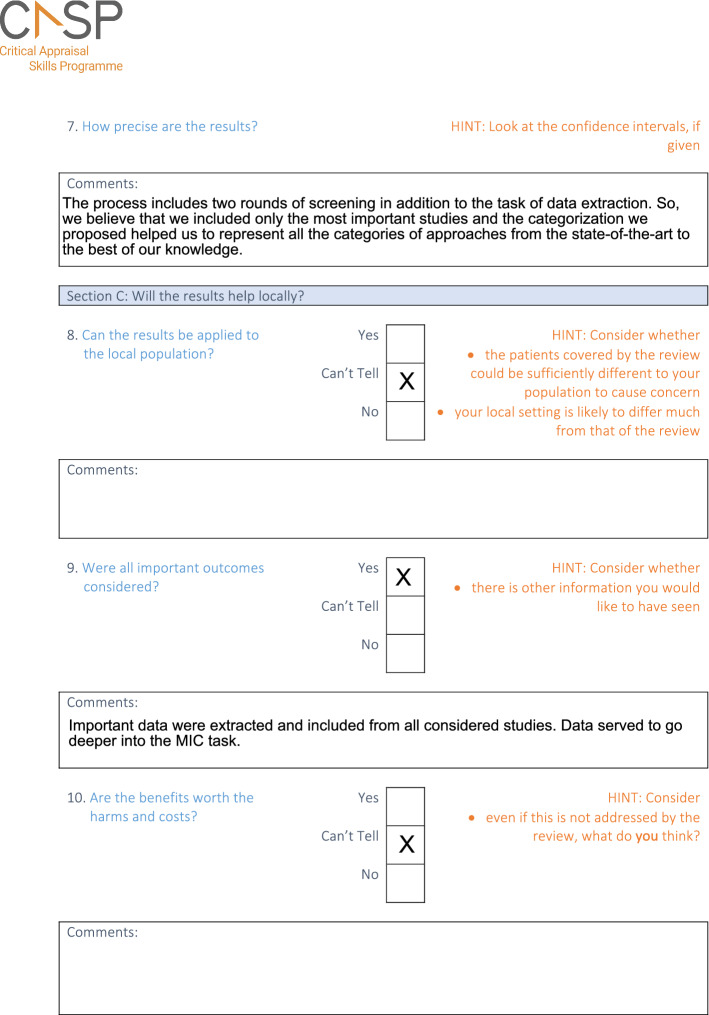
CASP Systematic review Checklist (page3)

## Appendix A: CASP checklist for rapid review quality appraisal from (CASP 2021)

See Figs. 16, 17 and 18.

## Appendix B: Comparison of MIC related studies

See Table 12.

**Table 12:** Analysis of some state-of-the-art comprehensive studies on MIC

Paper	Year	Approaches								Imaging modality					Metrics		Inputs	Outputs	Datasets
		ED	ED+att	Merged	T+R	R+G	T+G	R	T	X-ray	Ultrasound	MRI	Fundus	Others	Automatic Eval	Human Eval			
Zeng et al. (2020b)	2020	+								+	+				+		Medical images	Diagnosis	IU X-ray + own created dataset
Xiong et al. (2019)	2019		+							+					+		Chest X-ray images	Medical report	IU X-ray dataset
Yuan et al. (2019)	2019		+							+					+		Chest X-ray images	Medical report	IU X-ray and ChexPert datasets
Han et al. (2021)	2021						+					+			+		Spinal images	Medical report	Own created dataset
Syeda-Mahmood et al. (2020)	2020							+		+					+		Chest X-ray images	Medical report	Own created dataset
Singh et al. (2019)	2019	+								+					+		Chest X-ray images	Medical report	IU X-ray dataset
Lyndon et al. (2017)	2017	+												+	+		Medical images	Description	imageCLEF dataset
Beddiar et al. (2021)	2021		+											+	+		Medical images	Description	imageCLEF dataset
Castro et al. (2021)	2021	+												+	+		Medical images	Description	imageCLEF dataset
Charalampakos et al. (2021)	2021							+						+	+		Medical images	Description	imageCLEF dataset
Wang et al. (2021)	2021								+					+	+		Medical images	Description	imageCLEF dataset
Tsuneda et al. (2021)	2021		+											+	+		Medical images	Description	imageCLEF dataset
Nicolson et al. (2021)	2021		+											+	+		Medical images	Description	imageCLEF dataset
Sun et al. (2019)	2019	+												+	+		Breast mammography images	Medical report	Own created dataset
Alsharid et al. (2019)	2019			+							+				+	+	Fetal ultrasound images	Description	Own created dataset
Li et al. (2018)	2018					+				+					+	+	Lateral and frontal chest X-ray images	Medical report	IU X-ray + ChexPert dataset
Huang et al. (2019)	2019		+							+					+		Chest X-ray images + patient’s indication	Medical report	IU X-ray dataset
Alsharid et al. (2020)	2020			+							+				+		Fetal ultrasound images + audio transcripted into text	Description	Own created dataset
Benzarti et al. (2021)	2021	+												+	+		Medical images	Diagnosis	PEIR dataset
Gajbhiye et al. (2020)	2020		+							+					+		Lateral and frontal chest X-ray images	Medical report	IU X-ray dataset
Park et al. (2020)	2020		+							+					+		Chest X-ray images	Medical report	IU X-ray dataset
Rodin et al. (2019)	2019		+							+					+		Lateral and frontal chest X-ray images + patient’s indication	Medical report	Mimic CXR dataset
Kisilev et al. (2015)	2015								+					+	+		Breast mammography images	Medical report	Own created dataset
Yin et al. (2019)	2019		+							+					+		Chest X-ray images	Medical report	IU X-ray dataset
Yang et al. (2020)	2020		+							+					+	+	Lateral and frontal chest X-ray images	Medical report	IU X-ray dataset
Li et al. (2019)	2019				+					+					+	+	Lateral and frontal chest X-ray images	Medical report	IU X-ray dataset
Tian et al. (2020)	2020		+							+					+		Chest X-ray images + patient’s indication and doctor’s findings	Medical report	IU X-ray dataset
Yang et al. (2021)	2021		+								+				+		Ultrasound images	Medical report	PEIR, BCD 2018 and own created dataset
Shin et al. (2016)	2016	+								+					+		Chest X-ray images	Annotation	IU X-ray dataset
Xue et al. (2018)	2018		+							+					+		Chest X-ray images	Medical report	IU X-ray dataset
Wang et al. (2020)	2020					+				+					+	+	Chest X-ray images	Medical report	IU X-ray + CX-CHR dataset
Harzig et al. (2020)	2020	+								+					+		Chest X-ray images	Medical report	IU X-ray dataset
Gu et al. (2019)	2019		+							+					+		X-ray chest images	Medical report	IU X-ray + own created dataset
Xue et al. (2019)	2019		+							+					+		Chest X-ray images	Medical report	IU X-ray dataset
Harzig et al. (2019)	2019								+					+	+		Endoscopic gastrointestinal videos	Medical report	KvasirV2 + Medico2018 datasets
Wang et al. (2019)	2019			+						+					+		Chest X-ray images	Medical report	IU X-ray dataset
Han et al. (2018)	2018						+					+			+		Spinal images	Medical report	Own created dataset
Hasan et al. (2018)	2018		+											+	+		Medical images	Description	imageCLEF dataset
Zeng et al. (2018)	2018	+									+				+		Ultrasound images	Description	Own created dataset
Wu et al. (2017)	2017	+											+		+		Fundus images	Diagnosis	DIARETDB0 dataset
Xu et al. (2019)	2019		+											+	+		Medical images	Description	imageCLEF dataset
van Sonsbeek et al. (2020)	2020		+							+					+		Chest X-ray images + patient’s indication	Diagnosis	Mimic CXR dataset
Wang (2019)	2019							+						+	+		Medical images	Description	imageCLEF + ROCO datasets
Spinks et al. (2019)	2019		+							+					+		Chest X-ray images	Diagnosis	Own created dataset
Xie et al. (2019)	2019						+			+					+		Lateral and frontal chest X-ray images	Description	IU X-ray dataset
Mishra et al. (2020)	2020			+									+		+		Fundus images	Desciption	Stare dataset
Ambati and Reddy Dudyala (2018)	2018	+												+	+		Medical images	Description	imageCLEF dataset
Chelaramani et al. (2020)	2020	+											+		+		Fundus images	Diagnosis	Own created dataset
Onita et al. (2020)	2020								+	+					+		Chest X-ray images	Description	PADChest dataset
Rahman et al. (2018)	2018			+										+	+		Medical images	Description	imageCLEF dataset
Pelka et al. (2017)	2017	+												+	+		Medical images	Description	imageCLEF dataset
Zeng et al. (2020a)	2020	+									+				+		Ultrasound images	Annotation	Own created dataset
Hasan et al. (2017)	2017		+											+	+		Medical images	Medical report	imageCLEF dataset
Kougia et al. (2019)	2019					+								+	+		Medical images	Description	imageCLEF dataset

ED : Encoder-decoder architecture    ED + att : ED with attention    Merged : Merged architecture    T : Template-based technique    R : Retrieval-based technique    T + R : Fusion of Template-based and Retrieval-based techniques    T + G : Fusion of Template-based and Generative-based techniques    G + R : Fusion of Generative-based and Retrieval-based techniques

Others : Not mentioned modality, other modalities, different modalities at once

Automatic eval : Automatic metrics such as BLEU, CIDEr, METEOR, keyword accuracy, ARS, ...etc.    Human eval : Human evaluation + refers to the adoption of the method, the modality or the metrics. However, inside inputs, outputs and datasets columns, it refers to the addition

## Footnotes

1 https://academictorrents.com/details/5a3a439df24931f410fac269b87b050203d9467d

2 https://stanfordmlgroup.github.io/competitions/chexpert/

3 https://physionet.org/content/mimic-cxr-jpg/2.0.0/

4 https://bimcv.cipf.es/bimcv-projects/padchest/

5 https://figshare.com/projects/nmi-wsi-diagnosis/61973

6 https://peir.path.uab.edu/library/

7 https://www.imageclef.org/2017/caption

8 https://www.imageclef.org/2018/caption

9 https://github.com/razorx89/roco-dataset
